# Liposomes as Carriers of Membrane‐Associated Proteins and Peptides for Mass Spectrometric Analysis

**DOI:** 10.1002/anie.202101242

**Published:** 2021-03-23

**Authors:** Melissa Frick, Christian Schwieger, Carla Schmidt

**Affiliations:** ^1^ Interdisciplinary Research Center HALOmem Charles Tanford Protein Center Institute for Biochemistry and Biotechnology Martin Luther University Halle-Wittenberg Kurt-Mothes-Strasse 3a 06120 Halle Germany

**Keywords:** liposomes, mass spectrometry, membrane mimetics, peripheral membrane proteins, protein–lipid interactions

## Abstract

Membrane proteins are key players of the cell. Their structure and the interactions they form with their lipid environment are required to understand their function. Here we explore liposomes as membrane mimetics for mass spectrometric analysis of peripheral membrane proteins and peptides. Liposomes are advantageous over other membrane mimetics in that they are easy to prepare, can be varied in size and composition, and are suitable for functional assays. We demonstrate that they dissociate into lipid clusters in the gas phase of a mass spectrometer while intact protein and protein–lipid complexes are retained. We exemplify this approach by employing different liposomes including proteoliposomes of two model peptides/proteins differing in size. Our results pave the way for the general application of liposomes for mass spectrometric analysis of membrane‐associated proteins.

## Graphical abstract

Proteoliposome carriers for mass spectrometric analysis of membrane‐associated proteins and peptides enable the investigation of lipid clusters, protein oligomers, and protein–lipid interactions. This approach is versatile, applicable to many peripheral membrane proteins/peptides, and can be linked with functional assays.

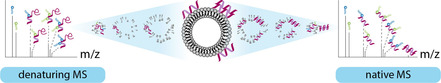

## Introduction

Membrane proteins represent more than a quarter of all cellular proteins. They are classified as integral or peripheral membrane proteins.^[1]^ While integral membrane proteins span the entire phospholipid bilayer or stably integrate into its hydrophobic core, peripheral membrane proteins only partially insert into the lipid membrane or interact with the lipid head groups. Due to their peripheral position and reversible attachment, they mediate between the aqueous environment of the cytosol and the hydrophobic membrane barrier. Accordingly, peripheral membrane proteins are involved in regulation of cell signalling, transportation of material to or from the membrane, as well as manipulation of the membrane's structure and organisation.

To understand the various functions of membrane proteins, their structural analysis as well as the characterization of the surrounding lipid environment and, in particular, the identification of specific protein–lipid interactions are of utmost importance. Traditional studies mostly targeted integral membrane proteins in contact with specifically bound lipids. In these studies, detergents are commonly used for purification and structural analysis;^[2]^ however, detergent micelles do not mimic a natural phospholipid bilayer.^[3]^ Therefore, a variety of membrane mimetics was developed providing a stable and native‐like environment for membrane proteins. Importantly, in addition to solubilisation of integral membrane proteins, some of these mimetics allow studying protein–membrane interactions of soluble proteins.

Despite their advantage over detergents, membrane mimetics contain additional phospholipids, detergents, scaffold proteins, or polymers and therefore still represent a challenge for classical structural techniques. Complementary techniques such as mass spectrometry (MS), and in particular native MS,^[4]^ proved useful alternatives for the analysis of integral membrane proteins.[^5^, ^6^, ^7^] This was initially achieved by dissociating the detergent micelle and releasing the free protein into the mass spectrometer.^[8]^ Later studies reconstituted membrane proteins in amphipols, bicelles, or various types of nanodiscs[^9^, ^10^, ^11^, ^12^] and showed that the native oligomeric states of the proteins[^10^, ^13^] as well as protein–lipid interactions[^13^, ^14^] are accessible by MS when using membrane mimetics. A recent breakthrough was the analysis of protein–ligand assemblies directly from native membrane vesicles.^[15]^

To study membrane association of peripheral membrane proteins, nanodiscs, and liposomes proved promising in recent studies.^[14]^ In particular, nanodiscs were employed to follow protein or peptide binding to various lipid membranes.[^16^, ^17^, ^18^] Here, we explore liposomes for the analysis of membrane‐associated proteins and peptides by MS. Liposomes are phospholipid bilayer vesicles which resemble natural phospholipid membranes. Due to their variability in size, composition, and amphiphilic character they are applicable to most membrane proteins. A great advantage of liposomes over other membrane mimetics is the possibility to perform functional assays such as transport through the membrane from the inner to the outer milieu or vice versa. In addition, protein binding to liposomes can be easily verified by flotation on a sugar gradient^[19]^ or co‐sedimentation.^[20]^ A combination of biochemical assays and subsequent MS analysis of the membrane test system, therefore, allows to directly link the functional and structural relationship of membrane proteins.

A first study assessed liposomes for MS analysis and showed that interactions between up to 100 lipid molecules can be preserved during native MS.^[21]^ Specificity of peptides towards specific lipids was also evaluated.[^21^, ^22^] Recently, we identified and quantified various phospholipids directly from the lipid bilayers of liposomes.^[23]^ In these experiments, using a conventional mass spectrometer under denaturing gas‐phase conditions, liposomes fully dissociated and individual lipids were maintained for subsequent analysis.

Here, we compare denaturing and native gas‐phase conditions to study binding of proteins and peptides to the phospholipid bilayer of liposomes. We envision that in the gas phase of a mass spectrometer, liposome membranes fully dissociate and membrane‐associated proteins or peptides are released. For this, we first complement our previous study by assessing liposome dissociation under native gas‐phase conditions. We then evaluate simultaneous detection of lipids and proteins/peptides in the same mass spectra. Finally, we study membrane binding of a model protein and a model peptide varying in size and lipid specificity. Namely, these are the p40(phox) domain of the human NADPH phagocyte oxidase complex specifically interacting with phosphatidylinositol‐3‐phosphates and the cationic and amphipathic peptide Melittin binding various lipid membranes and forming oligomers upon membrane association.

## Results and Discussion

### Experimental Design and Workflow

**Figure 1:**
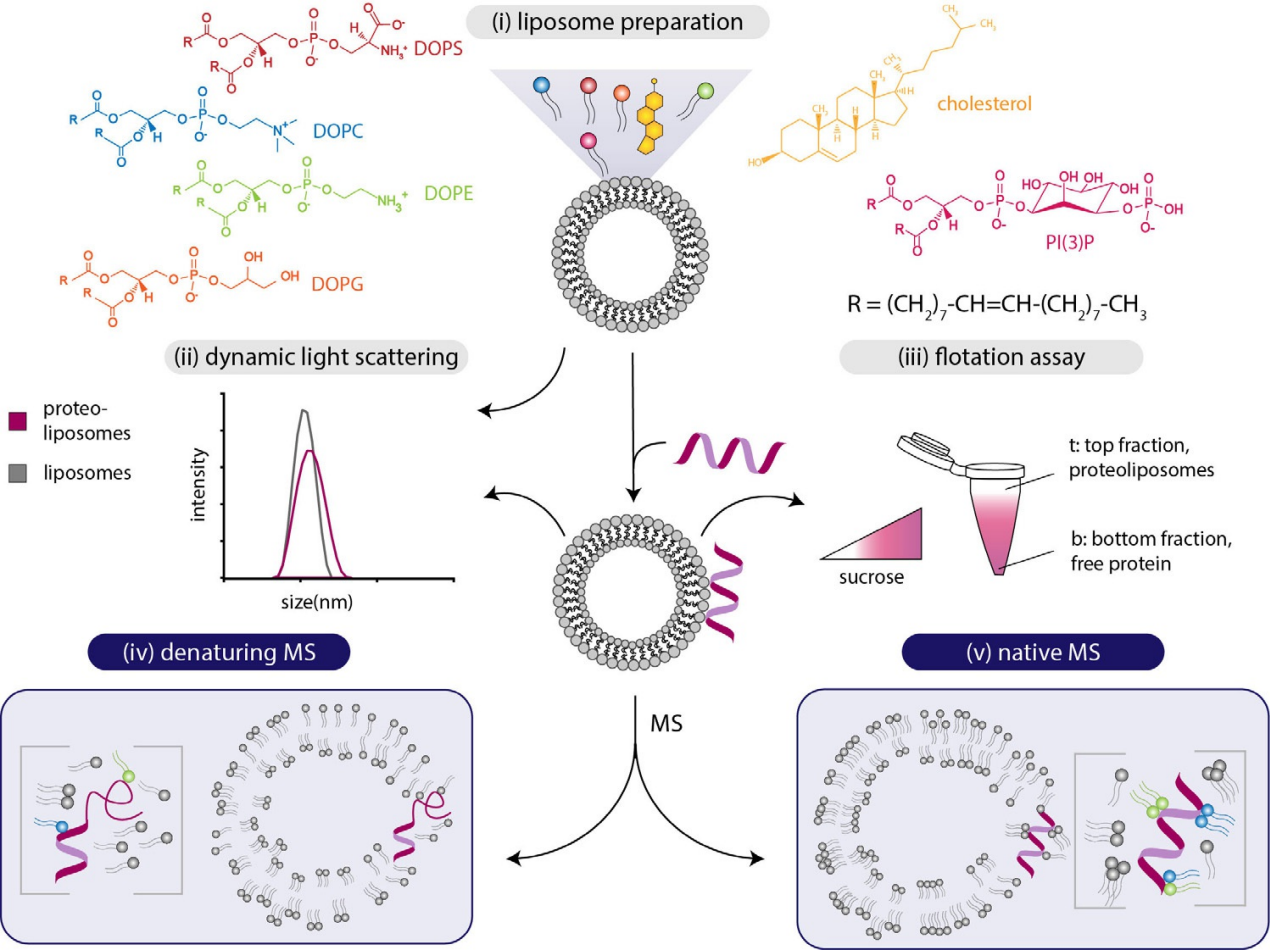
Workflow of liposome preparation (i) and analysis (ii–v). i) Liposomes composed of various lipids are prepared as described. Lipid structures are highlighted. ii) Liposomes or proteoliposomes are analysed by dynamic light scattering (DLS) determining the mean size distribution of the vesicles. iii) Liposomes and proteoliposomes are separated from free protein employing a sucrose density flotation assay. iv, v) Liposomes are analysed by MS iv) under denaturing or v) under native gas‐phase conditions.

We studied peripheral membrane proteins and peptides associated with the phospholipid bilayer of liposomes by MS. The experimental workflow is shown in Figure 1 and will be discussed in detail in the following section. First, we prepared liposomes of defined lipid composition resembling the natural target membrane of the proteins or peptides. Consequently, liposomes containing 1,2‐dioleoyl‐*sn*‐glycero‐3‐phosphocholine (DOPC), 1,2‐dioleoyl‐*sn*‐glycero‐3‐phosphoethanolamine (DOPE), 1,2‐dioleoyl‐*sn*‐glycero‐3‐phospho‐l‐serine (DOPS) and cholesterol at a molar ratio of DOPC/DOPE/DOPS/cholesterol 5:2:2:1 represent eukaryotic membranes.^[24]^ Liposomes supplemented with 1,2‐dioleoyl‐*sn*‐glycero‐3‐phospho‐(1′‐*myo*‐inositol‐3′‐phosphate) (PI(3)P), resulting in a lipid composition of DOPC/DOPE/PI(3)P 8:1:1, were utilised to study binding of the p40(phox) domain of the human NADPH phagocyte oxidase complex, which specifically associates with PI(3)P‐containing membranes.^[25]^ To mimic bacterial membranes, liposomes containing DOPE and 1,2‐dioleoyl‐*sn*‐glycero‐3‐phospho‐(1′‐*rac*‐glycerol) (DOPG) at a molar ratio of DOPE/DOPG 5:2 were employed.[^26^, ^27^] For liposome preparation, the lipids were mixed in the desired composition and dry lipid films were obtained after evaporation of the solvents. Unilamellar liposomes were then prepared by hydration in ammonium acetate solution followed by extrusion through a polycarbonate membrane of defined pore size (Figure 1, step i). Homogeneity and diameter of the resulting liposomes were confirmed by dynamic light scattering (DLS, Figure 1, step ii). Proteoliposomes formed during incubation of unilamellar liposomes with the peptides or proteins of interest. Peptide/protein association with the liposome membrane was then verified by flotation of the liposomes on a sucrose gradient. For this, proteoliposomes are overlaid with a sucrose gradient and, after centrifugation, liposomes and proteoliposomes float on top of the sucrose gradient, while free protein remains at the bottom. The presence of peptide/protein in the top (proteoliposomes) or bottom (free peptide/protein) fractions was then visualised by gel electrophoresis (Figure 1, step iii). For subsequent MS analysis, only those preparations of proteoliposomes were used in which the majority of the peptide/protein (i.e., >95 % binding as visualised by gel electrophoresis) associated with the liposome membranes.

MS experiments were performed using two instrumental set‐ups: (i) We employed a conventional Q Exactive Plus Hybrid‐Quadrupole‐Orbitrap mass spectrometer which is commonly used for lipid^[28]^ or peptide^[29]^ identification. The conditions in this mass spectrometer are rather harsh; non‐covalent interactions are hardly preserved and gas‐phase conditions are expected to be denaturing, that is, complete desolvation of the analytes is expected causing unfolding of proteins (Figure 1, step iv). Note that, contrary to expectations, non‐covalent association of co‐eluting peptides was recently described for this specific instrument.^[30]^ (ii) We also employed a Q‐ToF Ultima mass spectrometer modified for transmission of high‐mass complexes, thereby preserving non‐covalent interactions (Figure 1, step v).^[31]^ This type of mass spectrometer is commonly applied in native MS and allows the analysis of intact protein complexes and their interactions with ligands including lipids.^[4]^ By analysing proteoliposomes under denaturing and native MS conditions, we explore the applicability of liposomes to serve as carriers of membrane‐associated proteins and peptides into the gas phase of a mass spectrometer. We hypothesize that, following dissociation of the liposomes in the gas phase, intact peptides or proteins are released for MS analysis. Under denaturing gas‐phase conditions, liposome dissociation is supposedly facilitated, while native MS, on the other hand, allows maintaining non‐covalent protein and protein–lipid interactions.

### Intact Liposomes Dissociate into Lipid Clusters

**Figure 2:**
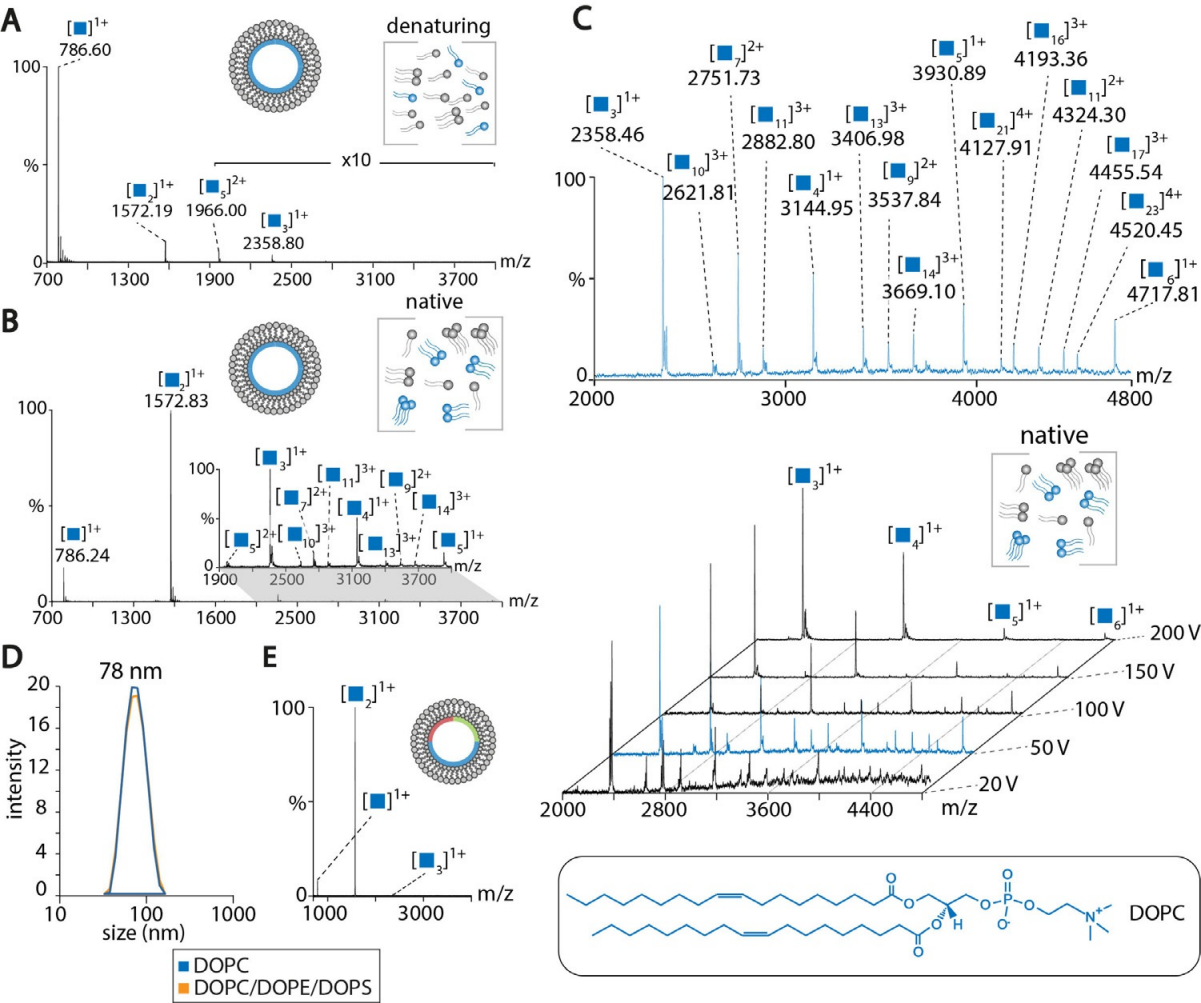
Mass spectra of liposomes. A) DOPC liposomes. Mass spectrum acquired on a Q Exactive mass spectrometer. B) DOPC liposomes. Mass spectrum acquired on a Q‐ToF mass spectrometer modified for native MS. Collisional voltage: 150 V. See Table S2 for *m*/*z* values. C) DOPC liposomes analysed by native MS at increasing collisional voltages (20–200 V). The spectrum at 50 V (blue) is magnified and lipid clusters are assigned. Note that some lipid clusters overlap. D) DLS of DOPC (blue) and mixed (orange) liposomes. E) Native MS of DOPC/DOPE/DOPS liposomes (molar ratio 5:2:2). Collisional voltage, 150 V.

For proof of principle, we first targeted “empty” liposomes varying in composition and size (Table S1). Figure 2 A shows the mass spectrum of DOPC liposomes acquired on the Q Exactive mass spectrometer. As expected, liposomes dissociate and the major signal observed at *m*/*z* 786.6 corresponds to monomeric DOPC confirming that the unmodified Q Exactive mass spectrometer provides indeed a denaturing gas‐phase environment. Small clusters of up to five lipid molecules were also observed albeit at very low intensities. Note that, in the Q Exactive mass spectrometer, ions do not pass through a collision cell on their way to the Orbitrap detector. When compared with a Q‐ToF mass spectrometer, collisional voltages cannot be applied without selection of specific ions in the quadrupole.

We then employed a Q‐ToF mass spectrometer modified for native MS and first analysed DOPC liposomes at high collisional voltage to provide conditions similar to the Q Exactive mass spectrometer. Intense peaks were observed for small, singly‐charged DOPC clusters containing up to three lipid molecules. Larger, singly, doubly, or triply charged lipid clusters containing up to 14 lipid molecules were observed at lower intensities (Figure 2 B). When compared with the denaturing conditions provided by the Q Exactive mass spectrometer, the major species is the DOPC dimer suggesting that non‐covalent interactions are promoted using this modified Q‐ToF mass spectrometer. To monitor dissociation of lipid clusters in the collision cell of the mass spectrometer, we applied increasing collisional voltages ranging from 20 to 200 V. As expected, higher collisional voltages cause dissociation of DOPC lipid clusters (Figure 2 C). At low collisional voltages, significantly larger, multiply charged clusters were observed; of these, clusters containing up to 23 lipid molecules could be assigned. Similar mass spectra were obtained for liposomes composed of DOPE (Figure S1A) and DOPS (Figure S1B).

Next, we prepared mixed liposomes containing DOPC, DOPE, and DOPS lipids at a molar ratio of 5:2:2 resembling a natural eukaryotic plasma membrane.^[24]^ DLS confirmed a homogeneous size distribution with a diameter of approx. 75 nm which is comparable with single‐component DOPC liposomes (Figure 1 D). Surprisingly, the native mass spectrum only showed DOPC clusters (Figure 1 E). Clusters of low abundant lipids were not observed.

We also prepared mixed liposomes containing cholesterol, a typical component of natural membranes which modulates membrane fluidity, elasticity, permeability, and curvature.^[32]^ The mass spectrum of liposomes composed of DOPC, DOPE, DOPS, and cholesterol at a molar ratio of 5:2:2:1, again, predominantly showed small DOPC clusters as well as low‐intense mixed DOPC/DOPE clusters (Figure S1C,D). Note that these clusters did not contain DOPS molecules; we assume that, due to the different head group structure, DOPS is excluded from cluster formation. Lipid clusters containing cholesterol were also not obtained suggesting that uncharged lipid components are not incorporated into phospholipid clusters during electrospray ionisation.

All liposomes analysed in these experiments were prepared at a total lipid concentration of 0.5 mm and extruded through a polycarbonate membrane with 50 nm pore size (Table S1). To test whether lipid concentration or liposome size affect the composition of lipid clusters, we increased the lipid concentration to 5 mm (Figure S1E) and the liposome size to a diameter of 100 nm (Figure S1F). With the exception that monomeric DOPC showed slightly higher intensities, no differences were observed for these liposomes (Figures S1D–F).

### Proteins and Lipids are Detectable in the Same Mass Spectrum

**Figure 3:**
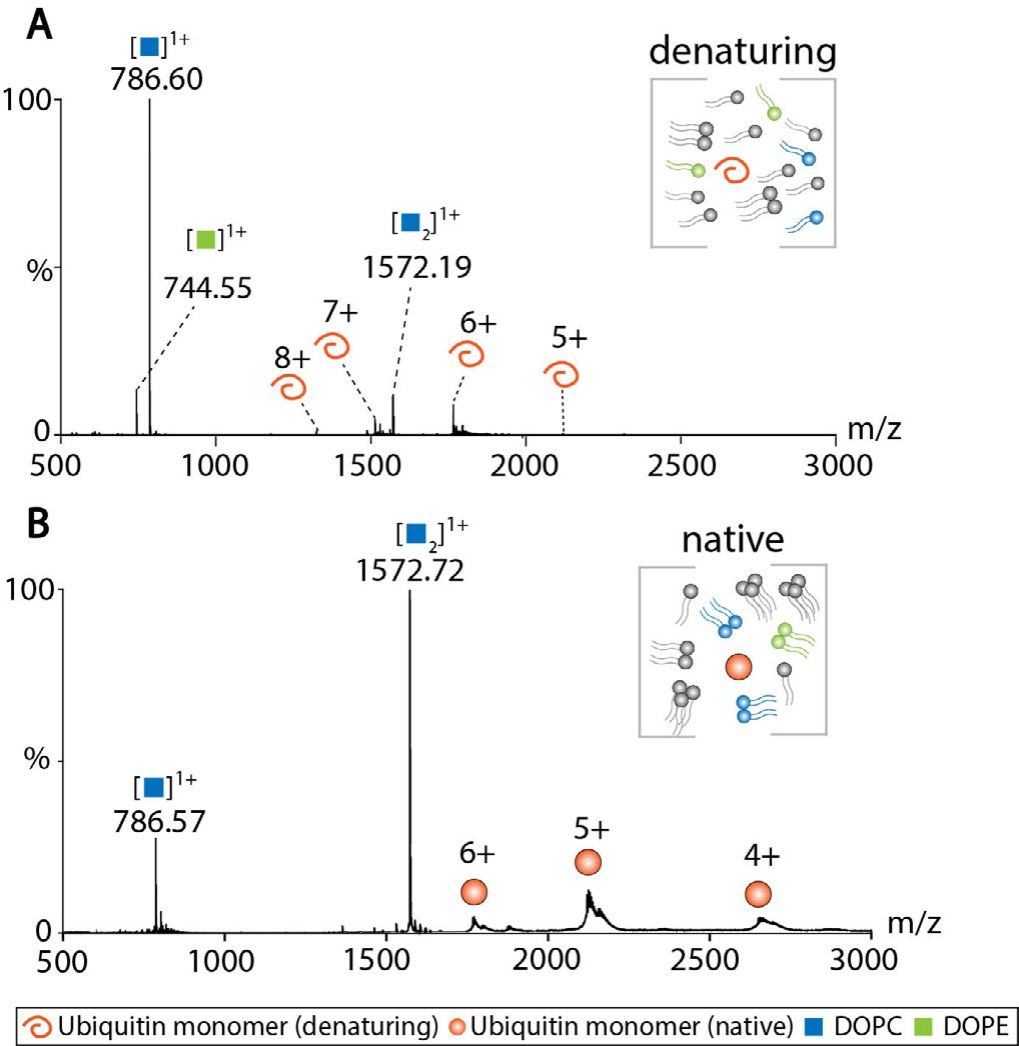
DOPC/DOPE/DOPS/cholesterol liposomes mixed with Ubiquitin. A) Mass spectrum acquired under denaturing gas‐phase conditions. DOPC and DOPE signals as well as a charge state series for monomeric Ubiquitin are assigned. B) Native mass spectrum of liposomes mixed with Ubiquitin. Charge states of monomeric Ubiquitin as well as DOPC clusters are assigned. Collisional voltage: 50 V. See legend for colour scheme and symbols.

Since liposomes dissociate in the gas phase of the mass spectrometer under denaturing gas‐phase conditions or at medium to high collisional voltages under native gas‐phase conditions, we next investigated whether proteins or peptides can be detected in the presence of liposomes without suppression of peptide/protein ions due to high intense lipid signals. For this, liposomes were mixed with soluble peptides or proteins, which do not interact with the employed phospholipids or biological membranes in general. The liposome‐peptide/protein mixtures were subsequently analysed by MS and resulting mass spectra were inspected for peptide/protein signals. We first chose Ubiquitin, a small protein of approx. 10 kDa, and mixed it with DOPC/DOPE/DOPS/cholesterol liposomes. Under denaturing conditions, that is, employing the Q Exactive mass spectrometer, a series of charge states ranging from 5+ to 8+ of Ubiquitin was observed. High intense lipid signals for singly charged DOPC and DOPE lipids were also detected (Figure 3 A).

Using native MS, we again found dimeric DOPC being the most intense species. Charge states of 4+ to 6+ corresponding to monomeric Ubiquitin were also obtained (Figure 3 B). This agrees well with the individual protein studied by denaturing and native MS in the absence of liposomes (Figure S2). Comparing denaturing and native gas‐phase conditions, we observe higher Ubiquitin charge states under denaturing conditions suggesting unfolding of the protein. The lower charge states observed in the native mass spectrum, on the other hand, indicate a more folded conformation. In addition, as also observed by native MS of liposomes alone, lipid interactions are preserved as indicated by the high intensity of the DOPC dimer (*m*/*z* 1572.72, Figure 3 B). Protein–lipid interactions were not observed.

We also analysed the peptide Angiotensin I, which has a similar molecular weight as the lipids used in our study. For this, Angiotensin I was mixed with DOPC/DOPE/DOPS/cholesterol liposomes and following incubation analysed by MS under denaturing and native gas‐phase conditions. Similar to Ubiquitin, we mainly observed monomeric and dimeric lipid clusters. Under both conditions, we also observed a signal corresponding to the peptide. Under denaturing gas‐phase conditions, we again observed a higher charge state (2+) than under native conditions (1+) (Figure S3A,B) suggesting that an unfolded conformation of the peptide is investigated while native MS preserves non‐covalent interactions even of a small peptide. This finding is supported by the mass spectra of the peptide acquired in the absence of liposomes (Figure S3C,D).

In summary, we found that peptides/proteins are detectable in the presence of lipids. However, due to high‐intense lipid signals, peptide/protein signals are comparably low in both denaturing and native mass spectra. Nonetheless, charge states are indicative of folded or unfolded conformations suggesting that protein and possibly protein–lipid interactions can be maintained and directly analysed by native MS. Protein–lipid interactions were not observed in the mass spectra of peptide/protein‐liposome mixtures confirming that unspecific interactions formed during the ionisation process can be excluded.

### MS of Proteins Associated with Liposome Membranes

Our overall aim was the direct MS analysis of peripheral membrane peptides or proteins associated with liposome membranes. For a first proof of principle, we chose the p40(phox) domain of the human NADPH phagocyte oxidase complex, which specifically associates with PI(3)P‐containing membranes through a binding pocket.[^25^, ^33^] After expression in *Escherichia coli*, the protein was purified through an affinity tag (see Supporting Information for details). Cleavage of the affinity‐tag was confirmed by gel electrophoresis and a molecular weight of 16.5 kDa was determined by denaturing and native MS (Figure S4).

**Figure 4:**
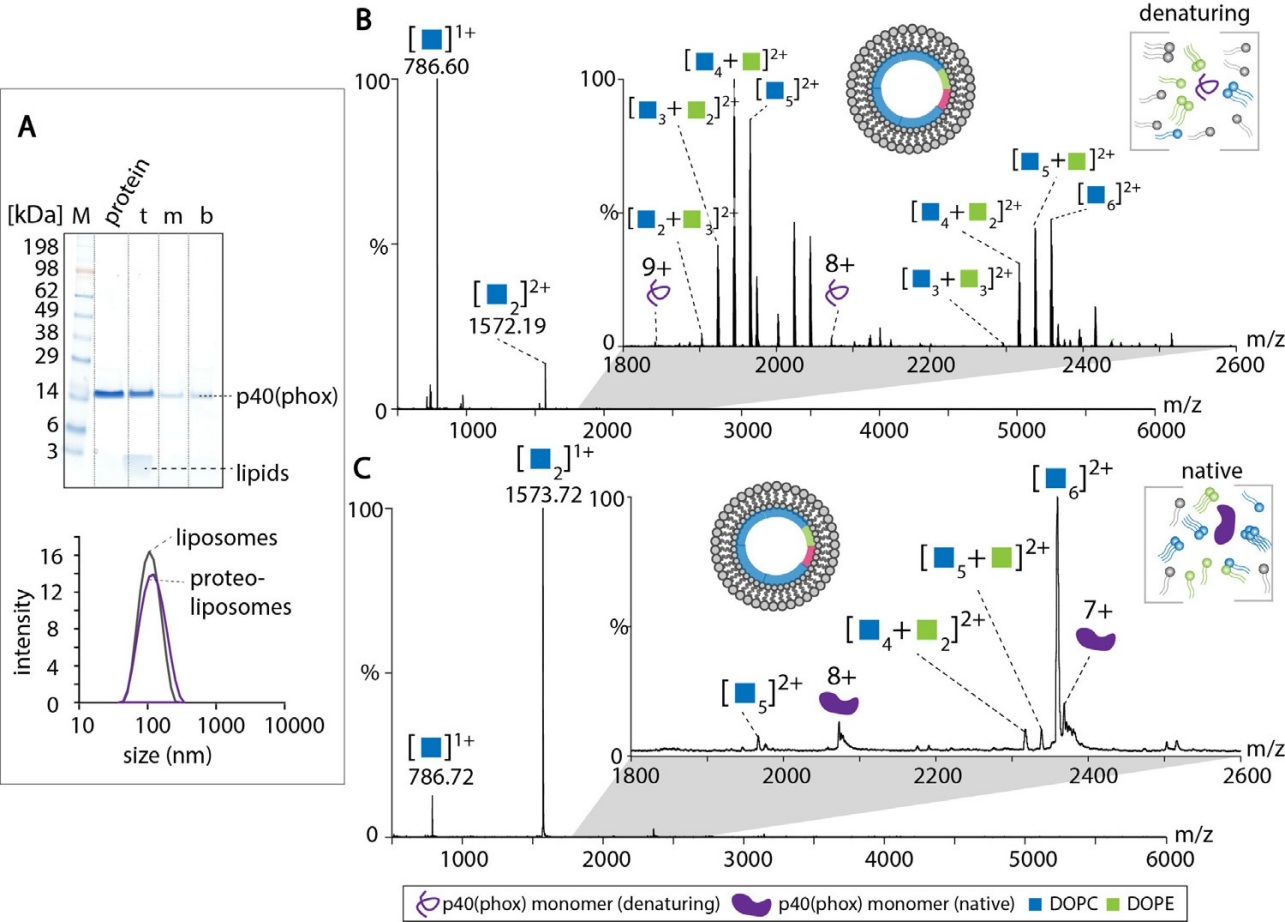
p40(phox) associated with DOPC/DOPE/PI(3)P liposomes. A) Liposome flotation assay and DLS analysis of p40(phox) liposomes. Top (t), middle (m), and bottom (b) fractions of the sucrose gradient were evaluated by gel electrophoresis. Size distributions of proteoliposomes and “empty” liposomes were compared by DLS showing a distribution of a diameter of around 100 nm. B) MS analysis of p40(phox) liposomes under denaturing conditions. DOPC and DOPE clusters as well as charge state series for monomeric p40(phox) are assigned. C) Native mass spectrum of p40(phox) liposomes. Lipid clusters of DOPC and DOPE as well as charge state series of monomeric p40(phox) are assigned. Collisional voltage: 50 V. See legend for colour scheme and symbols.

We first incubated p40(phox) with DOPC/DOPE/PI(3)P liposomes (molar ratio 8:1:1) and, as a control, with DOPC/DOPE liposomes (molar ratio 8:2). As expected, binding of p40(phox) was only observed for liposomes containing PI(3)P (Figure S5A). However, p40(phox) was rated a weak binder in a previous study.^[25]^ For subsequent analysis, we therefore performed a gel filtration step to separate proteoliposomes from unbound protein. The fraction with the highest protein content was then analysed in further experiments (Figure S5B,C). First, liposome binding was verified by flotation on a sucrose gradient (Figure 4 A). Indeed, a large proportion (approx. 95 %) of p40(phox) was observed in the top fraction of the sucrose gradient confirming formation of proteoliposomes. DLS confirmed the presence of a homogeneous population comparable to liposomes without protein (Figure 4 A). We then analysed p40(phox)‐proteoliposomes under denaturing and native gas‐phase conditions. Similar to peptide/protein–liposome mixtures (see above), the denaturing mass spectrum shows intense signals for monomeric and dimeric DOPC (Figure 4 B). In addition, mixed lipid clusters of DOPC and DOPE containing up to 6 lipid molecules were observed. Importantly, albeit at low intensity, we also observed signals corresponding to the 8+ and 9+ charge states of p40(phox). Using native MS, similar results were observed (Figure 4 C). Again, dimeric DOPC was the highest species of the mass spectrum. In addition to low‐intense mixed lipid clusters, the native mass spectrum also showed two charge states of p40(phox), the 8+ and 7+ charge states. When compared with the denaturing mass spectrum, these charge states are lower and show adduct peaks, which most likely originate from insufficient desolvation during transfer into the gas phase. Lipid adducts were not observed in these mass spectra; this is most likely attributed to the weak interactions between p40(phox) and PI(3)P reported previously.^[25]^ We assume that liposome dissociation causes loss of p40(phox)–PI(3)P interactions.

Similar to our observation made for peptide/protein–liposome mixtures, we conclude that the charge states of the proteins are indicative of their folding state after dissociation of the liposomes. Accordingly, we observed higher charge states under denaturing gas‐phase conditions when compared with native gas‐phase conditions. Ionisation of lipids appears to be favoured during electrospray ionisation and signals of peptides/proteins are mostly observed at low intensity. Nonetheless, the analysis of proteins specifically associated with liposome membranes is possible.

### Protein and Protein–Lipid Interactions can be Maintained in the Gas Phase after Dissociation of Liposomes

Finally, we applied the same strategy to a model peptide which forms oligomers and associates with phospholipid membranes. For this, we chose the cationic and amphipathic peptide Melittin, which exhibits strong antimicrobial activity.^[34]^ The molecular weight of Melittin is approx. 2.8 kDa and its oligomer masses lie well within the same mass range explored above for peptide/protein–liposome mixtures and p40(phox). Melittin was first analysed in the absence of liposomes. Under denaturing gas‐phase conditions, the monomeric peptide was observed (Figure S6A). The native mass spectrum, however, revealed monomeric, dimeric, and trimeric Melittin (Figure S6B) in agreement with previous studies which reported oligomer formation in solution.[^35^, ^36^, ^37^] The previously reported tetramer was, however, not observed presumably due to incomplete oligomer formation or destabilisation of the oligomer.

**Figure 5:**
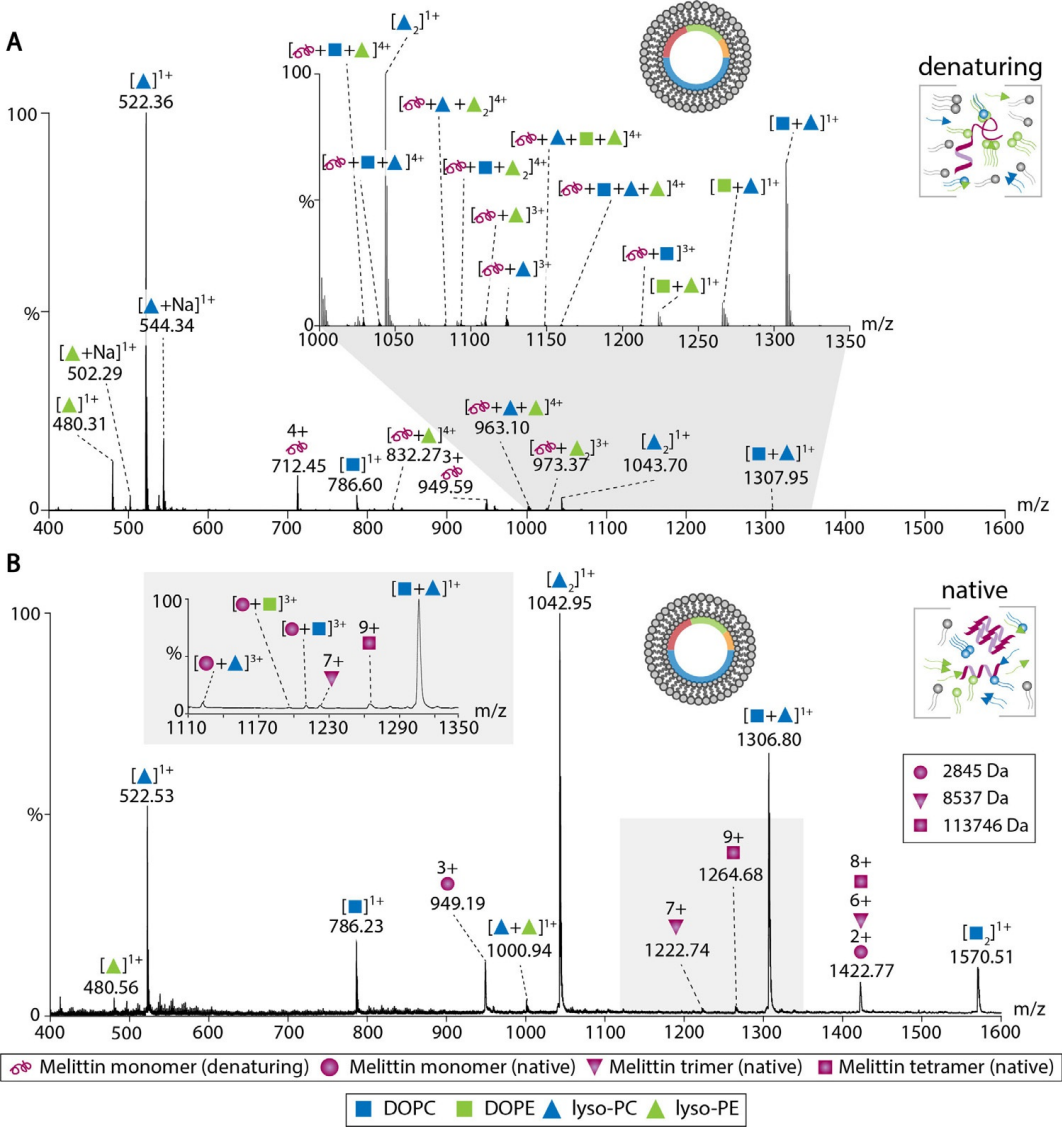
MS of Melittin proteoliposomes. A) Mass spectrum acquired on a Q Exactive Plus mass spectrometer under denaturing gas‐phase conditions. Monomeric Melittin, DOPC, DOPE, mixed lipid clusters, as well as protein–lipid complexes are assigned. See Table S5 for *m*/*z* values of the inset. B) Mass spectrum acquired on a Q‐ToF mass spectrometer modified for native MS at a collisional voltage of 50 V. The mass spectrum shows DOPC, DOPE, and mixed lipid clusters. Melittin oligomers up to tetramers were preserved. See Table S6 for *m*/*z* values of the inset. See legend for colour scheme and symbols.

Subsequently, Melittin was incubated with liposomes composed of DOPC/DOPE/DOPS/cholesterol at a molar ratio of 5:2:2:1. Melittin binding to the phospholipid bilayer was verified by liposome flotation (Figure S6C). DLS further confirmed a homogeneous liposome population of approx. 100 nm diameter with a slightly broader distribution after Melittin binding. Melittin proteoliposomes were first analysed under denaturing gas‐phase conditions. As expected, the mass spectrum shows monomeric Melittin as well as DOPC, DOPE, and mixed lipid clusters (Figure 5 A). Interestingly, we also observed high‐intense peaks corresponding in mass to lyso‐PC and lyso‐PE. These lyso‐lipids were likely obtained from cleavage with Phospholipase A2, a typical component in Melittin preparations from bee venom.^[35]^ Their origin was verified by incubation of liposomes with purified Phospholipase A2 followed by MS (Figure S7A,B) and tandem MS analysis (Figure S7C). Importantly, we identified Melittin interactions with lyso‐PC, lyso‐PE, DOPE, and DOPC, suggesting that peptide–lipid interactions were maintained even under denaturing gas‐phase conditions. A similar observation was previously made for peptides that co‐eluted during liquid chromatography and non‐covalently associated during MS analysis.^[30]^ We assume that electrostatic interactions between lipid head groups and amino acid side chains of Melittin are strong enough to be maintained under fairly harsh gas‐phase conditions.

We then investigated membrane‐bound Melittin by native MS. The mass spectrum reveals monomeric, trimeric, and tetrameric Melittin, lipid clusters, as well as protein–lipid complexes with DOPC and DOPE lipid species (Figure 5 B). Note that, when compared with the mass spectra acquired under denaturing conditions, only one associated lipid was observed with monomeric Melittin while the denaturing mass spectrum showed association of multiple lipids. We hypothesize that, under denaturing gas‐phase conditions, unfolding of the peptide during dissociation of the liposome membrane causes binding of additional lipid molecules in proximity. Accordingly, lipid interactions were only observed with the monomeric peptides in native MS measurements; we assume that the amino acid residues which mediate protein–lipid interactions are not accessible in the oligomeric structures. In addition, signals for preserved oligomers were of lower intensity and lipid adducts are most likely below the detection limit. Nonetheless, in contrast to denaturing MS, Melittin oligomers were preserved in these experiments.

Melittin also plays a role in the defence mechanism of honey bees against bacteria; therefore, we also analysed Melittin proteoliposomes composed of DOPE and DOPG lipids (molar ratio of 5:2) as a model of a bacterial membrane. Again, Melittin was incubated with preformed liposomes and membrane binding and homogeneity of the liposome population were verified by liposome flotation and DLS (Figure S6D). The mass spectrum acquired under denaturing gas‐phase conditions revealed monomeric Melittin, DOPE, and lyso‐PE as well as lyso‐PG at lower intensities (Figure S8A). The origin of lyso‐lipids was again verified by incubation of the liposomes with Phospholipase A2 (Figure S7D,E). Similar to the eukaryotic model membrane examined above, Melittin–lipid interactions with up to four bound lipids were preserved. Under native conditions, monomeric, dimeric, and trimeric Melittin were observed. However, only few protein–lipid interactions were recognised in these experiments (Figure S8B).

Using Melittin as a model peptide, we showed that both peptide–peptide and peptide–lipid interactions can be preserved when dissociating the liposome membrane of proteoliposomes under native gas‐phase conditions. Under denaturing conditions, however, peptide oligomers dissociate. Surprisingly, peptide–lipid interactions could be maintained under these harsh conditions suggesting that they are stable in the gas‐phase and are therefore likely ionic and polar interactions between lipid head groups and amino acid side chains.

In previous studies, Melittin oligomerisation and lipid interactions have been controversially discussed.[^16^, ^38^, ^39^] Accordingly, the oligomerisation states identified in our native MS experiments vary between the solubilised peptide and in the presence of different liposome membranes. For comparison, we, therefore, followed an independent approach and chemically cross‐linked Melittin in solution and associated with liposomes. For this, we incubated the peptide and proteoliposomes with bis(sulfosuccinimidyl)suberate and evaluated the presence of covalently linked oligomers by gel electrophoresis (Figure S9). Using increasing concentrations of the cross‐linker, we identified oligomers composed of an increasing number of Melittin monomers. Note that these oligomers exceed a well‐defined tetramer and rather unspecifically aggregate (Figure S9A). In the presence of liposomes, Melittin showed a lower oligomerisation propensity. Nonetheless, oligomers of at least trimers were clearly observed (Figure S9B). Unspecific oligomerisation states are in agreement with different models describing pore formation in the membrane. In these models, Melittin associates with the membrane and passes through different states before membrane pores are eventually formed.^[39]^ In addition, membrane binding, oligomerisation, and pore formation strongly depend on the concentration of the peptide as well as the peptide‐to‐lipid ratio.^[40]^ The peptide‐to‐lipid ratio explored here supports membrane binding and oligomerisation of Melittin, however, it is just above the desired ratio at which pore formation is initiated. We conclude that the oligomeric states captured in our analyses are intermediates of Melittin oligomerisation and pore formation.

Even though we observed intensive binding of Melittin to DOPC, DOPE, and even lyso‐lipids thereof, we did not observe interactions with DOPS or DOPG in any of the acquired mass spectra. We therefore evaluated Melittin–lipid interactions in two independent experiments. First, we performed liposome flotation assays using single‐component liposomes of DOPC and DOPG. The assays were performed as described above and Melittin binding was assessed by gel electrophoresis (Figure S10). Similar to our MS analyses, we identified strong binding to DOPC‐liposomes (Figure S10A); however, Melittin binding to DOPG liposomes was significantly weaker as indicated by the presence of the peptide in the middle fraction of the sucrose gradient (Figure S10B). The presence of Melittin in the middle fraction of these liposome flotation assays suggests that DOPG‐bound Melittin dissociates from the liposome membrane during gradient centrifugation.

We, therefore, further investigated lipid binding specificity and monitored binding of Melittin to various lipid monolayers over time (Figure S11). Recorded binding curves show a high affinity for a zwitterionic DOPC monolayer. Binding to negatively charged DOPG and DOPS monolayers was, on the contrary, substantially weaker (Figure S11A–C). However, the curve shape suggests remodelling of the negatively charged lipid monolayers after injection of Melittin presumably due to ionic interactions between the peptide and the lipid head groups. By plotting the surface pressure increase caused by Melittin binding as a function of the initial surface pressure of the lipid monolayer, the synergy factor is obtained from the slope of the linear dependency (see Methods and Figure S11D–F). The high synergy factor calculated for DOPC clearly reflects the high binding affinity of Melittin while the low synergy factor for incorporation of Melittin into DOPS and DOPG monolayers reveal only moderate affinity for these lipids (Figure S11G). In addition, we determined the maximum insertion pressure (MIP), that is the maximal lipid density at which the monolayer is still being penetrated by the peptide. A markedly higher MIP of DOPC as compared to DOPS and DOPG indicates again a higher incorporation tendency of Melittin into zwitterionic lipid layers (Figure S11H).

These findings are in agreement with our MS experiments revealing strong affinity for DOPC (and DOPE) but not for DOPS or DOPG lipids. Some previous studies controversially suggest preferred binding of Melittin to neutral (zwitterionic) or anionic lipids.[^16^, ^41^, ^42^, ^43^] In agreement with our results, a recent study showed that Melittin electrostatically interacts with anionic lipids, however, insertion into the membrane is driven by the hydrophobic effect.^[39]^ We therefore conclude that Melittin interacts with anionic lipids, however, stably associates with zwitterionic lipids. Nonetheless, one should keep in mind that natural membranes contain a variety of different lipids and individual lipid components contribute to membrane attraction, association, insertion, and permeabilisation.

## Conclusion

In summary, we show that liposomes dissociate in the gas‐phase of the mass spectrometer under denaturing and native gas‐phase conditions. Even though lipid signals are of high intensity, peptides and proteins are detectable in the same mass spectrum and their charge states are indicative of a denatured or folded conformation. Importantly, lipid clusters, protein oligomers, and strong protein–lipid interactions are preserved when using a mass spectrometer modified for native MS. Independent biophysical approaches verified our findings, making liposomes a considerable alternative to study membrane‐associated proteins and peptides as well as protein/peptide–lipid interactions in future.

## Conflict of interest

The authors declare no conflict of interest.

## Supporting information

As a service to our authors and readers, this journal provides supporting information supplied by the authors. Such materials are peer reviewed and may be re‐organized for online delivery, but are not copy‐edited or typeset. Technical support issues arising from supporting information (other than missing files) should be addressed to the authors.

Supplementary
