# Supplementary material for: Liposomes as Carriers of Membrane‐Associated Proteins and Peptides for Mass Spectrometric Analysis
Source: Angew Chem Int Ed Engl. 2021 Mar 23;60(20):11523–30. doi: 10.1002/anie.202101242 (PMC8252038; doi:10.1002/anie.202101242)
Supplement: Supplementary file 1 — Supplementary [file ANIE-60-11523-s001.pdf]

Angewandte Chemie
Eine Zeitschrift der Gesellschaft Deutscher Chemiker

Supporting Information

Liposomes as Carriers of Membrane-Associated Proteins and Peptides for Mass Spectrometric Analysis

*Melissa Frick, Christian Schwieger, and Carla Schmidt**

anie_202101242_sm_miscellaneous_information.pdf

Materials and methods

Materials

1,2-dioleoyl-*sn*-glycero-3-phosphocholine (DOPC), 1,2-dioleoyl-*sn*-glycero-3-phospho-L-serine (DOPS), 1,2-dioleoyl-*sn*-glycero-3-phosphoethanolamine (DOPE), 1,2-dioleoyl-*sn*-gylcero-3-phospho-(1'-rac-glycerol**)** (DOPG), cholesterol (ovine) and 1,2-dioleoyl-*sn*-glycero-3-phospho-(1'-myo-inositol-3'-phosphate) (PI(3)P) were purchased from Avanti Polar Lipids Inc. (Alabaster, AL, USA). Ubiquitin, Angiotensin I, Melittin and Phospholipase A2 were purchased from Sigma Aldrich. The p40(phox) plasmid was gifted by Brett Collins (Addgene plasmid # 119123; http://n2t.net/addgene:119123; RRID: Addgene_119123). Bis(sulfosuccinimidyl)suberate (BS3) was purchased from Thermo Scientific.

Recombinant protein expression and purification of p40(phox)

The plasmid encoding the p40(phox) domain of the human NADPH phagocyte oxidase complex fused with a GST-tag was transformed in BL21 (DE3) *E.coli* cells and expressed in TB Media at 37°C. The culture was induced with 1mM IPTG and cells were grown overnight at 25°C. Protein purification was performed using a Glutathione Sepharose High performance column (Cytiva, USA). The protein was eluted with 50 mM HEPES, 150 mM NaCl, 10 mM glutathione and 0.1 mM TCEP, pH 7.4. The GST-tag was cleaved by dialysis overnight using 100 U Thrombin. Subsequently, the protein solution was subjected to a reverse Glutathione-Sepharose column (Cytiva, USA) and eluted with 20 mM HEPES, supplemented with 150 mM NaCl and 0.1 mM TCEP, pH 7.4. The p40(phox) domain was then subjected to size-exclusion chromatography using a High load Superdex 16/600 column pre-equilibrated with 50 mM HEPES, 150 mM NaCl and 0.5 mM TCEP, pH 7.4. The purified protein was concentrated to 1.5 mg/ml using an Amicon Ultra 10 kDa centrifugal filter (Millipore, USA). The concentration of the protein was determined by Nanodrop (DeNovix®, USA).

Preparation of liposomes

Lipids were dissolved in chloroform and mixed in varying proportions (see **Table S1**). The solvent was evaporated using a rotary evaporator. Multilamellar vesicles were obtained by hydration of the lipid film in 200 mM ammonium acetate at room temperature for approx. 1 h. Unilamellar vesicles were then prepared by extrusion of multilamellar vesicles. For this, 21 strokes through a polycarbonate membrane (Whatman, Nuclepore Track-Etch Membrane) of defined pore size were performed using a mini-extruder (Avanti polar lipids, Alabaster, AL, USA). See **Table S1** for details on pore sizes.

Preparation of Proteoliposomes

For protein/peptide binding to liposome membranes, approx. 1.5 mg/ml p40(phox) and 1 mg/ml Melittin, respectively, in 200 mM ammonium acetate were incubated with liposomes (1.5 mM - 2.5 mM) for 1h at room temperature at a protein-lipid molar ratio of 1:50 (Melittin, p40(phox)) and 1:100 (p40(phox)). To verify protein association to the liposomes, proteoliposomes were subjected to a liposome flotation assay. For this, a sucrose gradient from 0 M (top) to 1.2 M (bottom) was prepared as follows: Proteoliposomes were mixed with 2.5 M sucrose in 200 mM ammonium acetate buffer yielding a concentration of 1.2 M sucrose (bottom layer). 0.75 M sucrose in 200 mM ammonium acetate (middle layer) and 200 mM ammonium acetate (top layer) were layered on top. The established gradient was centrifuged at 268,000 × g for 2 h or overnight at 22°C. Bottom, middle and top fractions were collected manually and analysed by gel electrophoresis[1].

Gel electrophoresis

Proteins were separated by gel electrophoresis using the NuPAGE system (Thermo Fisher Scientific) according to manufacturer's protocols. Briefly, samples were loaded onto 4-12 % Bis-Tris protein gels. The SeeBlue Plus2 Pre-stained Protein standard (Themo Fisher Scientific) was used as molecular weight marker. Proteins were separated at 200 V for 30 min. Protein gels were stained with Coomassie using InstantBlue Protein Stain solution (Expedeon). The gel was subsequently washed with water.

Dynamic light scattering

Measurements were performed using 70 µl of the liposome sample (see Table S1) in a micro UV cuvette (Brand GmbH & CoKG, Wertheim, Germany) using a Zetasizer Nano S instrument (Malvern Instruments, Worcestershire, UK). The particles were irradiated with a helium-neon laser of 633 nm and 4 mW, scattering angle 173 °. The following parameters were applied: material, liposomes; dispersant, water; temperature, 22°C; microcuvette, ZEN0040 – disposable micro cuvette. Average values of liposome and proteoliposome hydrodynamic diameters were obtained from size distribution histograms.

Denaturing Mass Spectrometry

Liposomes were analysed by denaturing mass spectrometry using a commercially available Q-Exactive Plus Hybrid Quadrupole-Orbitrap Mass Spectrometer (Thermo Fisher Scientific). For this, 2-4 µl of sample were loaded into a borosilicate offline emitter coated with gold/palladium (Thermo Fisher Scientific). Mass spectrometric settings were: electrospray capillary voltage, 1.7 kV; capillary temperature, 250 °C; MS resolution, 70.000; and RF-lens level, 50. Mass spectra were recorded in positive ion mode. Tandem MS analysis was

performed at different collisional energies ranging from 10 - 40 NCE. The MS scan range varied for different measurements (500-6000 *m/z*).

Native Mass Spectrometry

Proteins and liposomes were analysed by native mass spectrometry using a Q-ToF Ultima modified for transmission of high masses[2]. For this, gold-coated glass capillaries were prepared in-house[3]. 2-3 µl of the sample were loaded into the emitters for each analysis. Mass spectrometric settings were: capillary voltage, 1.5 – 1.7 kV; cone voltage, 80 V; RF lens voltage, 80V; collision energy, 20 - 200 V. Mass spectra were processed using MassLynx 4.0 and analysed using Massign software[4].

Chemical cross-linking

35 µM of Melittin and Melittin-proteoliposomes were cross-linked with varying amounts of BS3 (35 µM up to 875 µM) for 1h at 25 °C and 350 rpm in a thermocycler. Covalently liked Melittin oligomers were visualized by gel electrophoresis as described above.

Monolayer adsorption method

Monolayer adsorption experiments were performed using a deltaPi-4x Tensiometer (Kibron Inc., Helsinki, Finland) consisting of 4 Wilhelmy type pressure sensors installed over 4 circular Langmuir troughs. Each trough was filled with 1.1 mL Phosphate buffered saline (PBS) as subphase. The troughs were thermostated at 20 °C using an external circulating water bath and the whole set-up was enclose within a PMMA cover to protect the surfaces from dust deposition and reduce evaporation. The purity of the subphase as checked by recording a baseline at π = (0 ± 0.1) mN/m for at least 30 min. Subsequently, lipids were spread at the air/buffer interphase by dropwise depositing lipid solutions in chloroform / methanol (3:1, vol/vol) until the desired initial surface pressure (π_0) was reached. The solvent was allowed to evaporate for at least 10 min and the lipid film was equilibrated for at least 30 min. Then, 11 µL of a Melittin solution (in PBS, 1 mg/mL) were injected though an injection port directly into the subphase, i.e. underneath the equilibrated lipid monolayer, resulting in a Melittin subphase concentration of c = 3.5 nM. To ensure homogeneous distribution of the peptide, the subphase was gently stirred during injection and throughout the measurement using magnetic stirring bars (6x3 mm). After injection, the surface pressure was recorded as a function of time until a constant value (π_{eq}) was reached (typically at 4 h). The surface pressure increase was calculated as $\Delta\pi = \pi_{eq} - \pi_0$. For each lipid species, this experiment was repeated at various π_0. $\Delta\pi$ was plotted as function of π_0 und fitted with a linear function $\Delta\pi = A\cdot\pi_0 + B$. The maximum insertion pressure (MIP) and the synergy factor of the lipid-peptide interaction were calculated from the fitting parameters as $MIP = -B/A$ and $synergy = A + 1$[5,6]. If the maximum insertion pressure (MIP) is ≥ 30 mN/m the peptide is considered to insert into self-assembled bilayers of the same lipid. The synergy is positive or negative for attractive or repulsive interactions between lipids and peptide, respectively.

Supplementary Figures

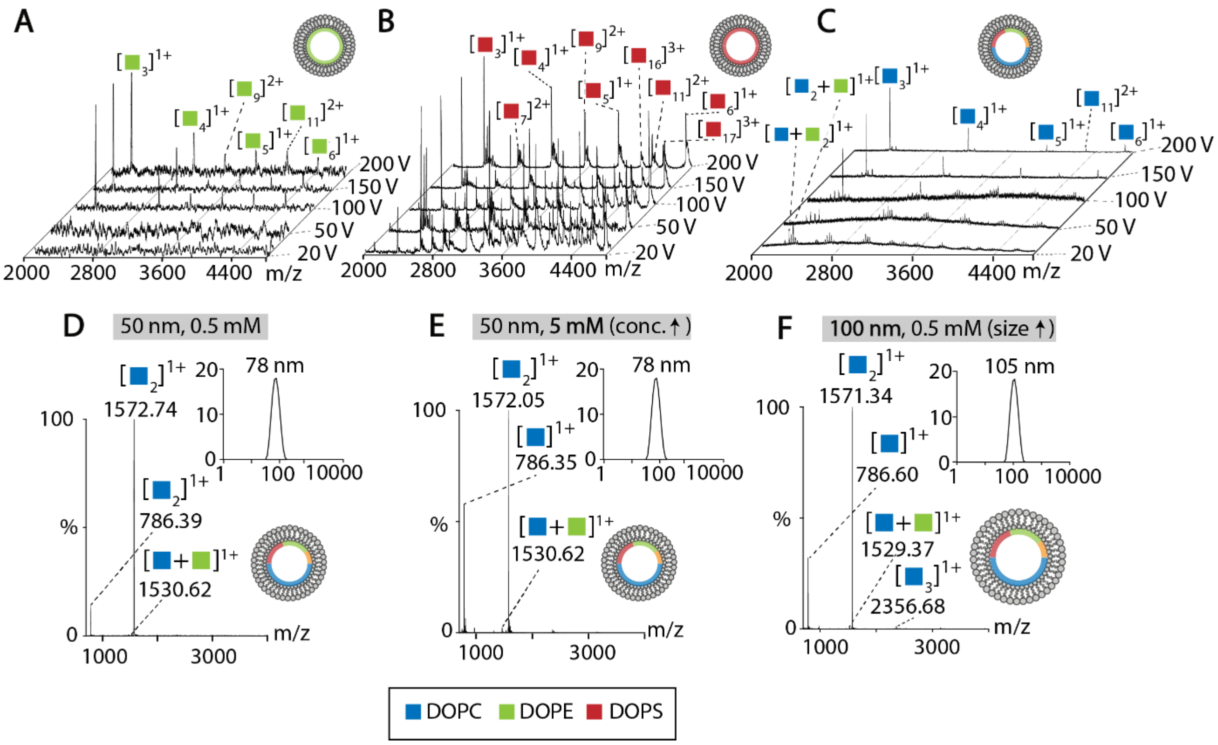

Figure S1. Native MS of liposomes varying in lipid composition. (A) DOPE liposomes. Collisional voltages ranging from 20-200 V. **(B)** DOPS liposomes. Collisional voltages ranging from 20-200 V. **(C)** DOPC/DOPE/DOPS/cholesterol liposomes (molar ratio 5:2:2:1). Collisional voltages ranging from 20-200 V. **(D)** DOPC/DOPE/DOPS/cholesterol liposomes (molar ratio 5:2:2:1). Collisional voltage: 150 V, concentration: 0.5 mM, pore size of the polycarbonate membrane: 50 nm. DLS reveals a mean liposome diameter of approx. 80 nm. **(E)** DOPC/DOPE/DOPS/cholesterol liposomes (molar ratio: 5:2:2:1). Collisional voltage: 150 V, concentration 5 mM, pore size of the polycarbonate membrane: 50 nm. DLS reveals a mean liposome diameter of approx. 80 nm. **(F)** DOPC/DOPE/DOPS/cholesterol liposomes (molar ratio: 5:2:2:1). Collisional voltage: 150 V, concentration 0.5 mM, pore size of the polycarbonate membrane: 100 nm. DLS reveals a mean liposome diameter of approx. 100 nm.

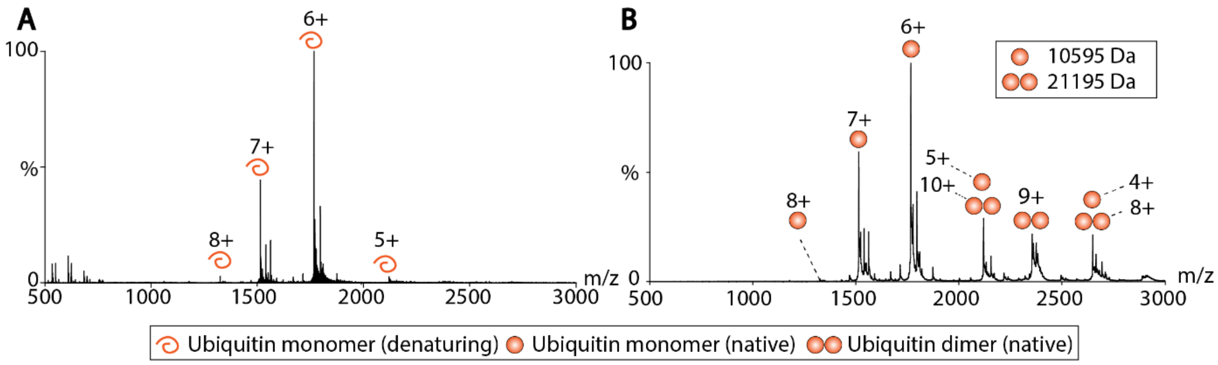

Figure S2. Mass spectra of Ubiquitin in the absence of liposomes. (A) Ubiquitin analysed under denaturing gas-phase conditions reveals a charge state series for monomeric Ubiquitin (5+ to 8+). **(B)** Mass spectrum of Ubiquitin under native gas-phase conditions. Charges state series corresponding to monomeric (4+ to 8+) as well as dimeric (8+ to 10+) Ubiquitin could be assigned. Collisional voltage: 50 CV. See legend for symbols.

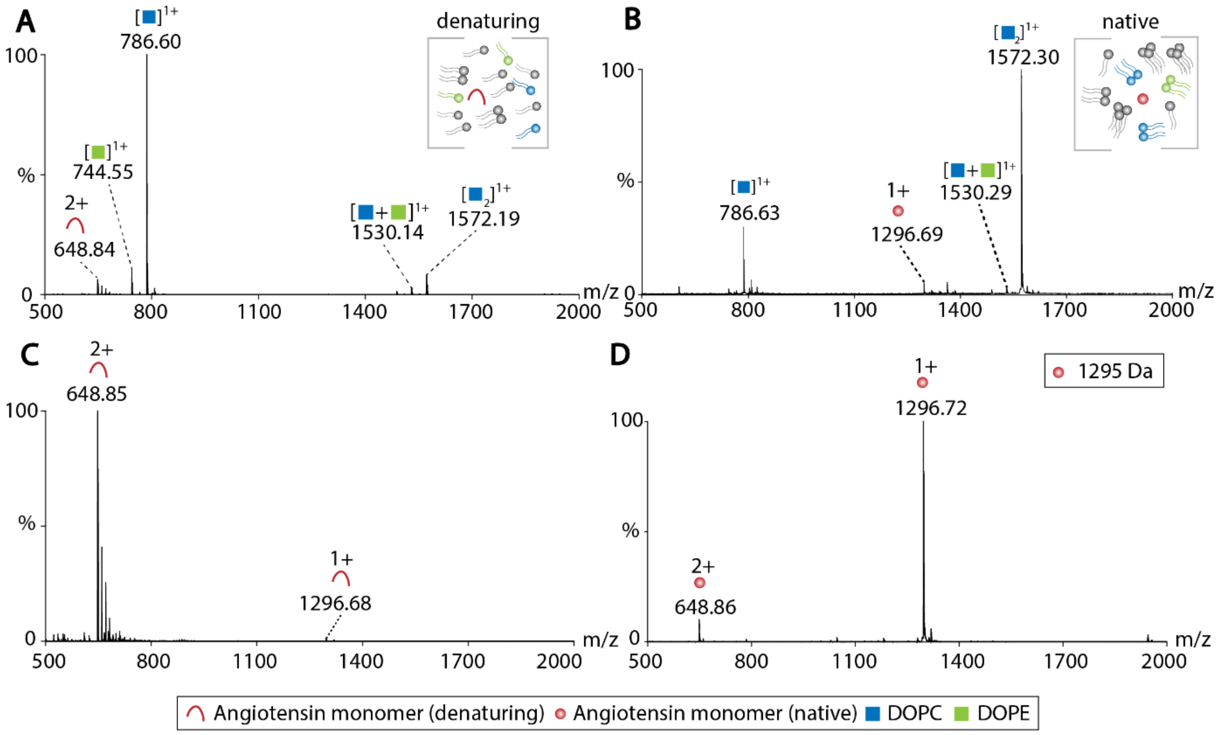

Figure S3. Mass spectra of Angiotensin I in the presence and absence of DOPC/DOPE/DOPS/cholesterol liposomes (molar ratio 5:2:2:1). (A) Mass spectrum of Angiotensin I mixed with liposomes under denaturing gas-phase conditions. The 2+ charge state of monomeric Angiotensin I, lipid clusters of DOPC as well as mixed clusters were assigned. **(B)** The native mass spectrum of Angiotensin I mixed with liposomes shows the 1+ charge state of monomeric Angiotensin I. Clusters of DOPC and mixed lipid clusters were also observed. Collisional voltage: 50 CV. **(C)** The mass spectrum of Angiotensin I under denaturing gas-phase conditions shows monomeric Angiotensin I. The 2+ charge state shows highest intensity. **(D)** The mass spectrum acquired under native gas-phase conditions shows monomeric Angiotensin I. The 1+ charge state shows the highest intensity. Collisional voltage: 50 CV. See legend for colour scheme and symbols.

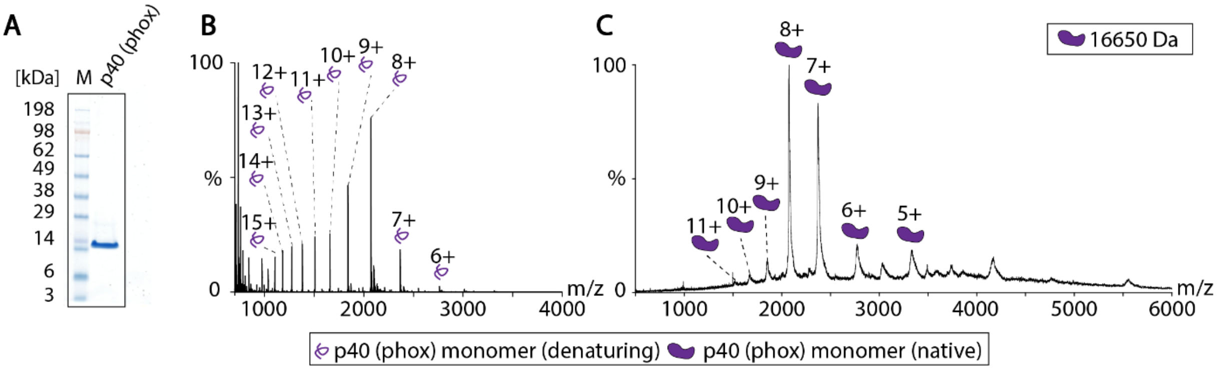

Figure S4. Gel electrophoresis and MS analysis of p40(phox). (A) SDS-PAGE of purified p40(phox). The molecular weight of approx. 16.5 kDa confirms the presence of p40(phox) omitting the GST-tag. **(B)** Mass spectrum of p40(phox) under denaturing gas-phase conditions showed a charge state series of monomeric p40(phox) (6+ to 18+). **(C)** Under native gas-phase conditions, a charge state series of monomeric p40(phox) with lower charge states was observed (5+ to 11+). Collisional voltage: 50 CV. See legend for symbols.

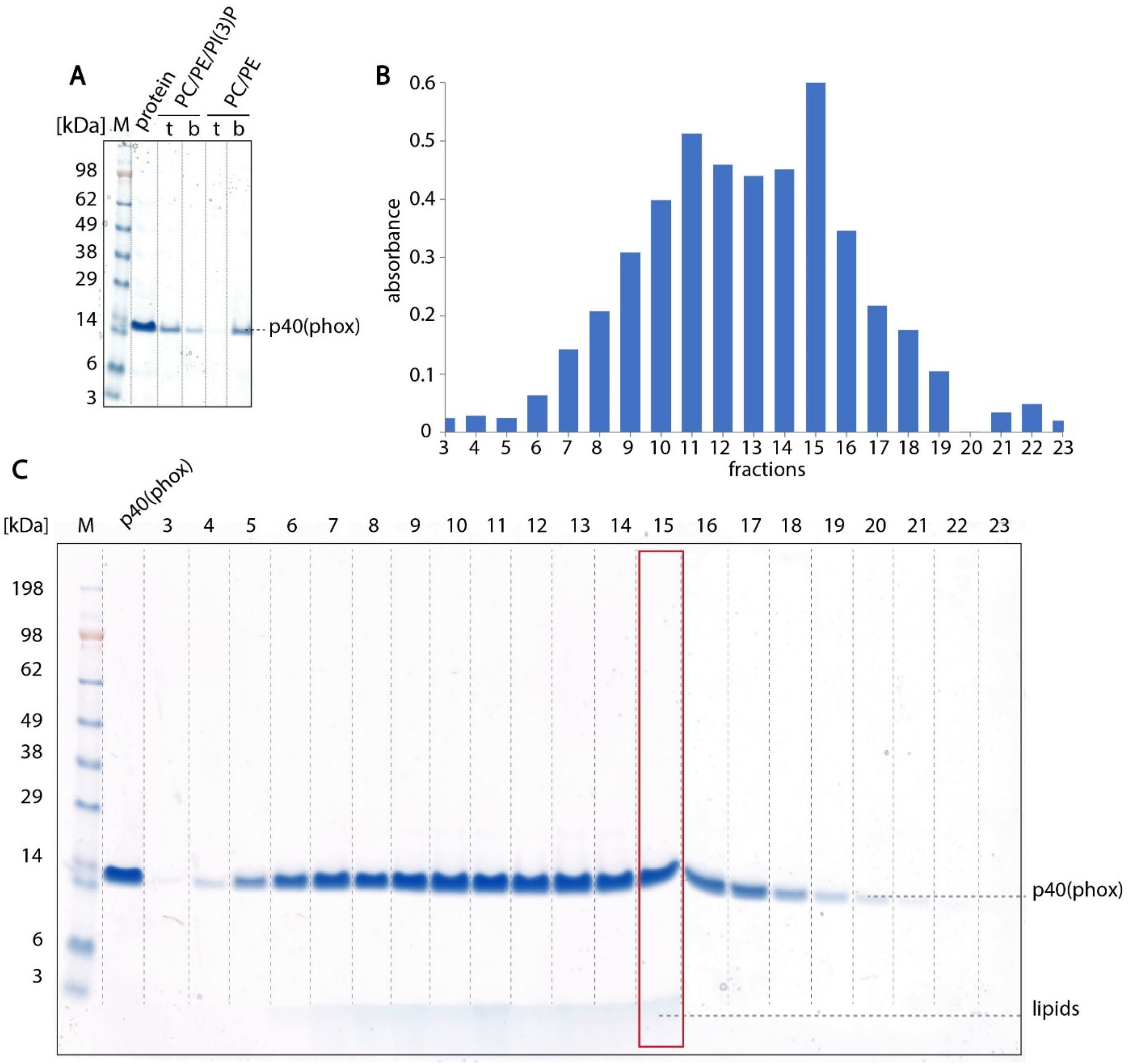

Figure S5: Flotation assay and gel filtration of p40(phox) proteoliposomes. (A) Liposome flotation assay of p40(phox) liposomes. Specific binding to PI(3)P containing membranes was confirmed by comparing DOPC/DOPE/PI(3)P liposomes with DOPC/DOPE liposomes. Top (t) and bottom (b) fractions of the sucrose gradient were evaluated by gel electrophoresis. **(B)** Fractions of the gel filtration column were collected dropwise and protein concentrations were determined by nanodrop. The absorbance of fractions 3 to 23 was plotted in a bar diagramme. **(C)** Fractions 3 to 23 of the gel filtration were visualized by SDS-PAGE. The molecular weight marker (M) indicates masses in kDa. P40(phox) was observed at approx. 16 kDa. Fraction 15 (red box) showed the highest protein content and was subjected to further analysis by DLS and MS. The presence of lipids in this fraction further confirmed the presence of liposomes.

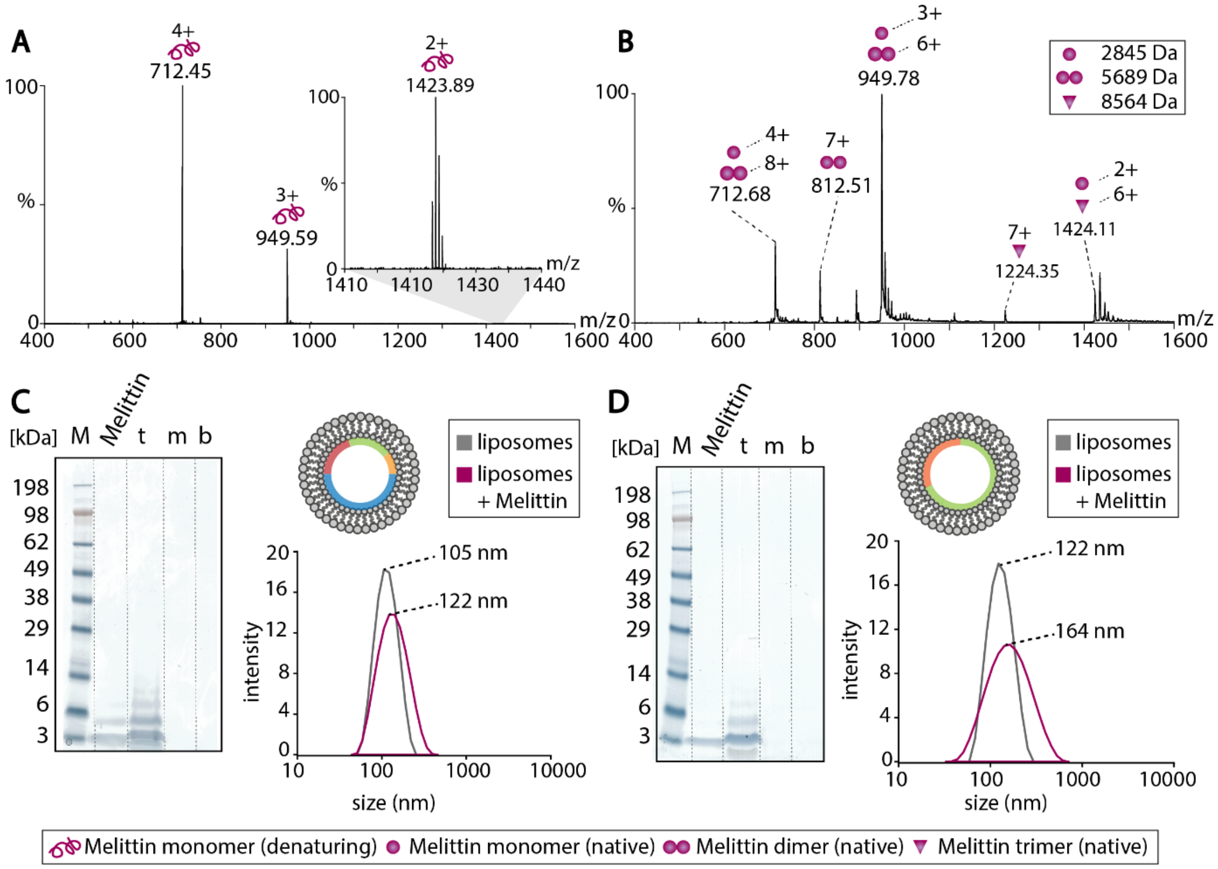

Figure S6. **MS analysis of Melittin and liposome flotation assays of Melittin proteoliposomes.** Top (t), middle (m) and bottom (b) fractions of the sucrose gradient were evaluated by gel electrophoresis (n=3). Size distributions of proteoliposomes and "empty" liposomes were compared by DLS (n=3). The molecular weight marker (M) indicates masses of proteins separated by gel electrophoresis in kDa. **(A)** Melittin analysed under denaturing gas-phase conditions reveals a peak series (2+ to 4+) corresponding to monomeric Melittin. **(B)** Under native MS conditions, peaks corresponding to monomeric, dimeric and trimeric Melittin were observed. Collisional voltage: 50 CV. **(C)** Melittin proteoliposomes composed of DOPC/DOPE/DOPS/cholesterol (molar ratio 5:2:2:1). **lhs:** Gel electrophoresis revealed the presence of Melittin in the top fraction of the sucrose gradient confirming binding to the liposome membrane. **rhs:** DLS of proteoliposomes showed a homogenous size distribution with a maximum at approx. 120 nm. For comparison, DOPC/DOPE/DOPS/cholesterol liposomes showed a size distribution maximum at approx. 105 nm. **(D)** Melittin proteoliposomes composed of DOPE/DOPG (molar ratio 5:2) **lhs:** Gel electrophoresis revealed the presence of Melittin in the top fraction of the sucrose gradient confirming binding to the liposome membrane. **rhs:** DLS of proteoliposomes showed homogenous size distribution with a maximum at approx. 160 nm. For comparison, DOPE/DOPG liposomes showed a size distribution of approx. 120 nm. See legend for colour scheme and symbols.

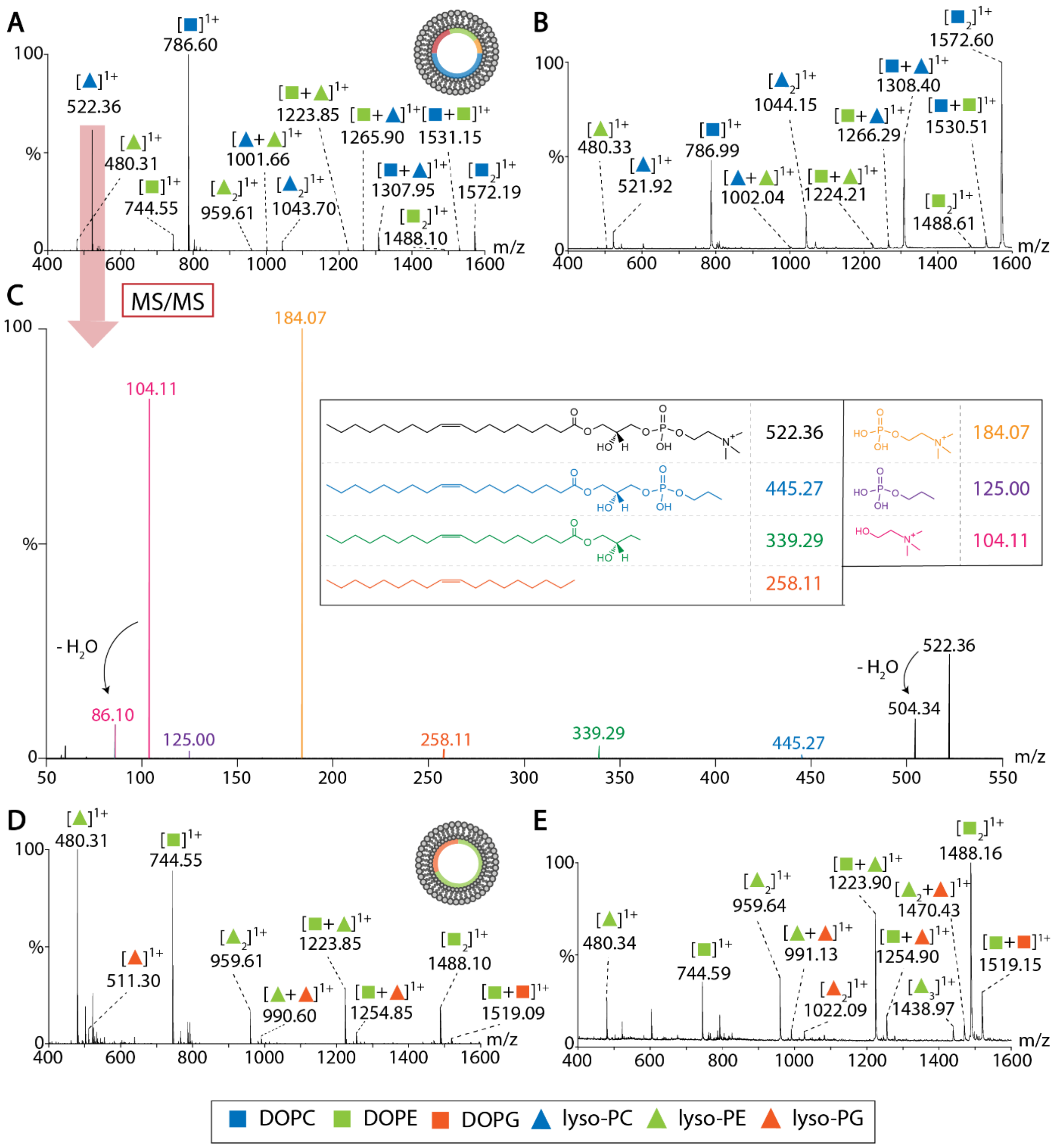

Figure S7. Liposomes of different compositions incubated with Phospholipase A2. (A) Mass spectrum of DOPC/DOPE/DOPS/cholesterol liposomes (molar ratio 5:2:2:1) under denaturing gas-phase conditions. DOPE, DOPC, lyso-PE and lyso-PC as well as lipid clusters thereof are assigned. **(B)** Native mass spectrum of DOPC/DOPE/DOPS/cholesterol liposomes. DOPE, DOPC, lyso-PE and lyso-PC as well as lipid clusters thereof are assigned. **(C)** Tandem mass spectrum (MS/MS) of lyso-PC (*m/z* 522.36). Fragments specific to the lipid head group and the acyl chain are highlighted. **(D)** Mass spectrum of DOPE/DOPG liposomes under denaturing gas-phase conditions. DOPE, DOPG, lyso-PE and lyso-PG as well as lipid clusters thereof are assigned. **(E)** Native mass spectrum of DOPE/DOPG liposomes. DOPE, DOPG, lyso-PE and lyso-PG as well as lipid clusters thereof are assigned. See legend for colour scheme and symbols.

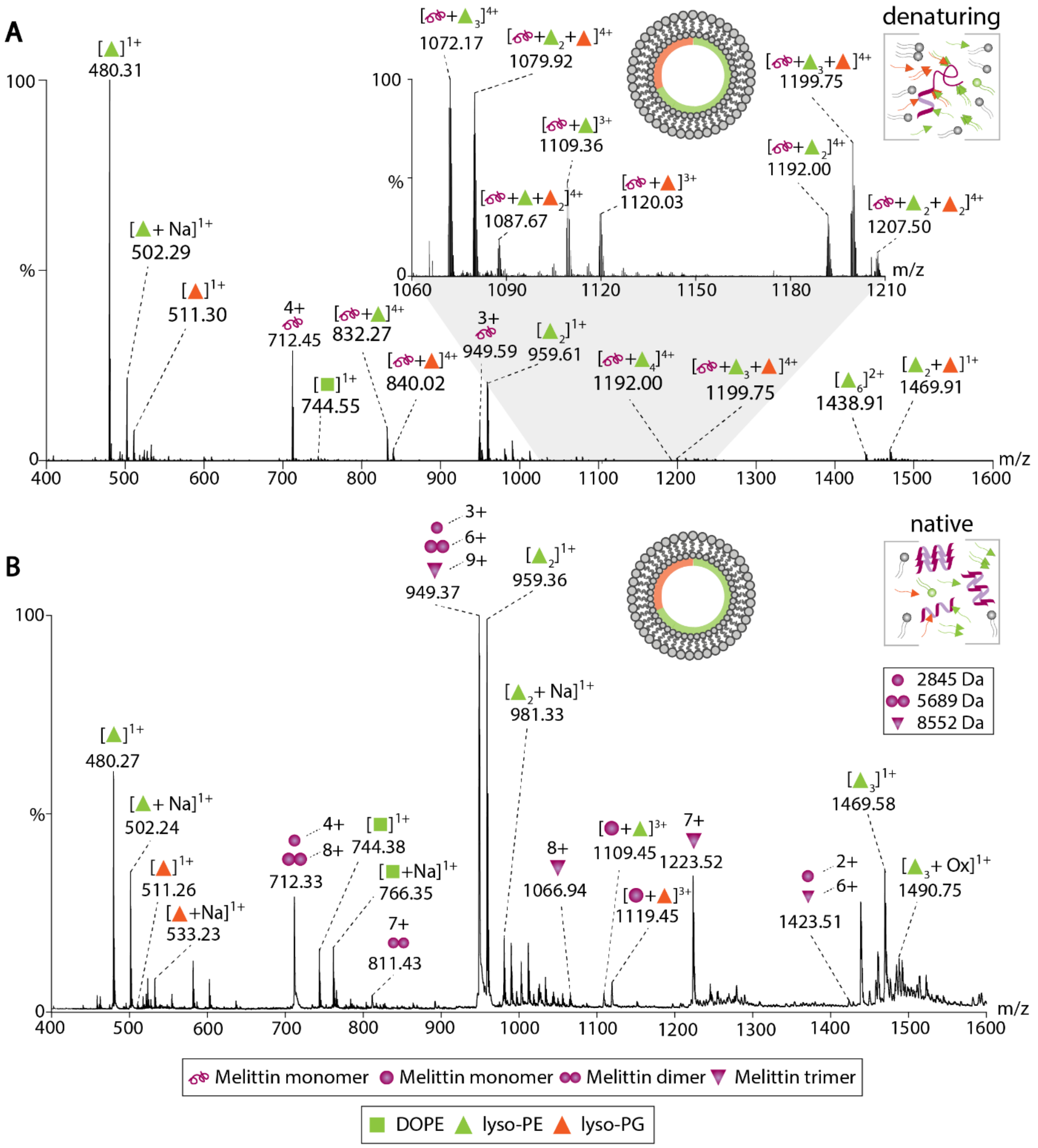

Figure S8. MS of Melittin proteoliposomes composed of DOPE/DOPG (molar ratio 5:2). (A) Mass spectrum of Melittin acquired under denaturing gas-phase conditions. Monomeric Melittin, DOPE, lyso-PE, DOPG, lyso-PG, mixed lipid clusters as well as protein-lipid complexes are assigned. **(B)** The native mass spectrum reveals individual lipids and mixed lipid-clusters. Melittin oligomers up to trimers could be preserved. Collisional voltage: 50 CV. See legend for colour scheme and symbols.

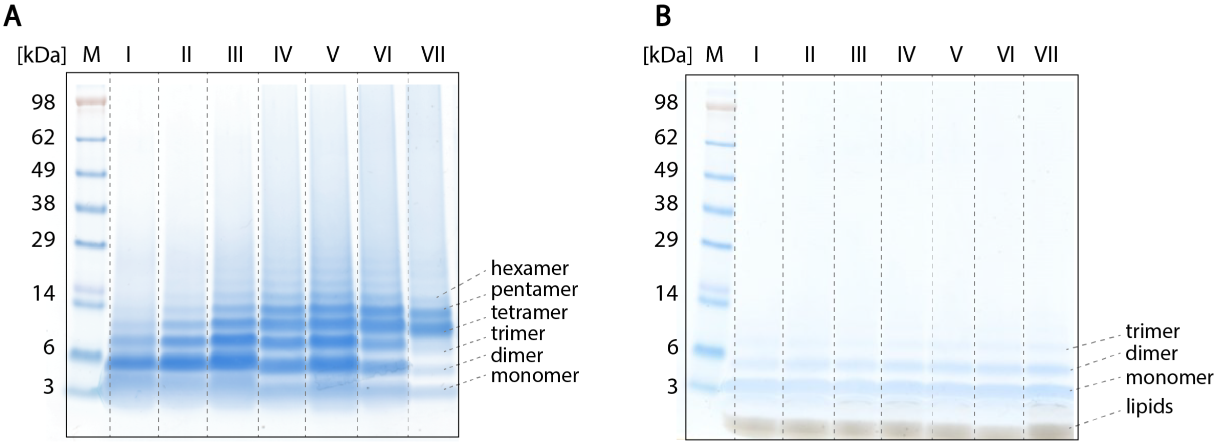

Figure S9. Chemical cross-linking of Melittin in solution and in Melittin proteoliposomes composed of DOPC/DOPE/DOPS/cholesterol (molar ratio 5:2:2:1). 35 µM Melittin was cross-linked with BS3 and covalently linked Melittin oligomers were visualized using gel electrophoresis. **(A)** 35 µM Melittin was loaded as a control (I). Samples containing 35 µM Melittin each were cross-linked with increasing amounts of BS3: 35 µM (II), 87.5 µM (III), 175 µM (IV), 262.5 µM (V), 350 µM (VI), 525 µM (VII), 875 µM (VIII) BS_3. Oligomers up to hexamers were observed. **(B)** 35 µM Melittin proteoliposomes were loaded as a control (I). 35 µM Melittin proteoliposomes were each cross-linked with increasing amounts of BS3: 35 µM (II), 87.5 µM (III), 175 µM (IV), 262.5 µM (V), 350 µM (VI), 525 µM (VII), 875 µM (VIII) BS_3. Oligomers up to trimers were observed.

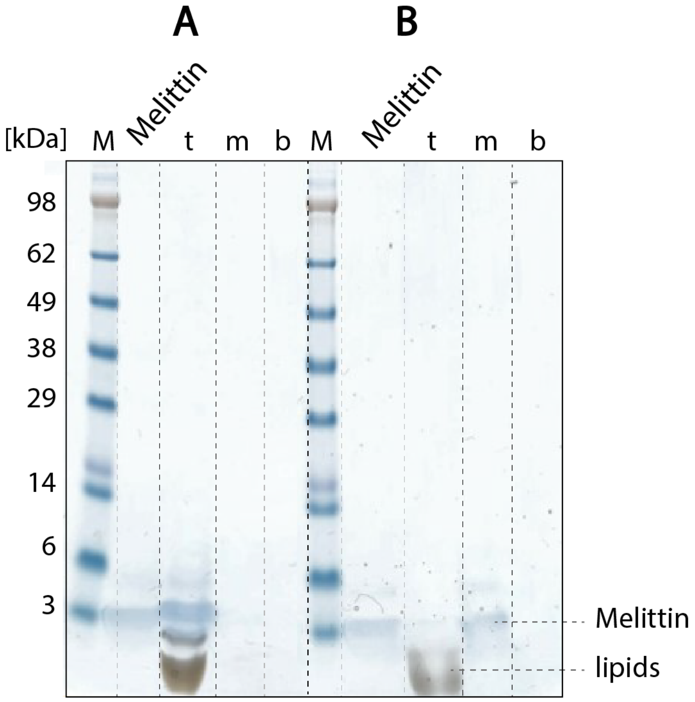

Figure S10. Liposome flotation assay to study binding of Melittin to single-component liposomes. Top (t), middle (m) and bottom (b) fractions of the sucrose gradient were evaluated by gel electrophoresis. **(A)** Melittin was incubated with DOPC-liposomes. Melittin was observed in the top fraction confirming binding to the liposome membrane. **(B)** Melittin was incubated with DOPG-liposomes. Melittin was only observed in the middle fraction suggesting dissociation of Melittin during centrifugation.

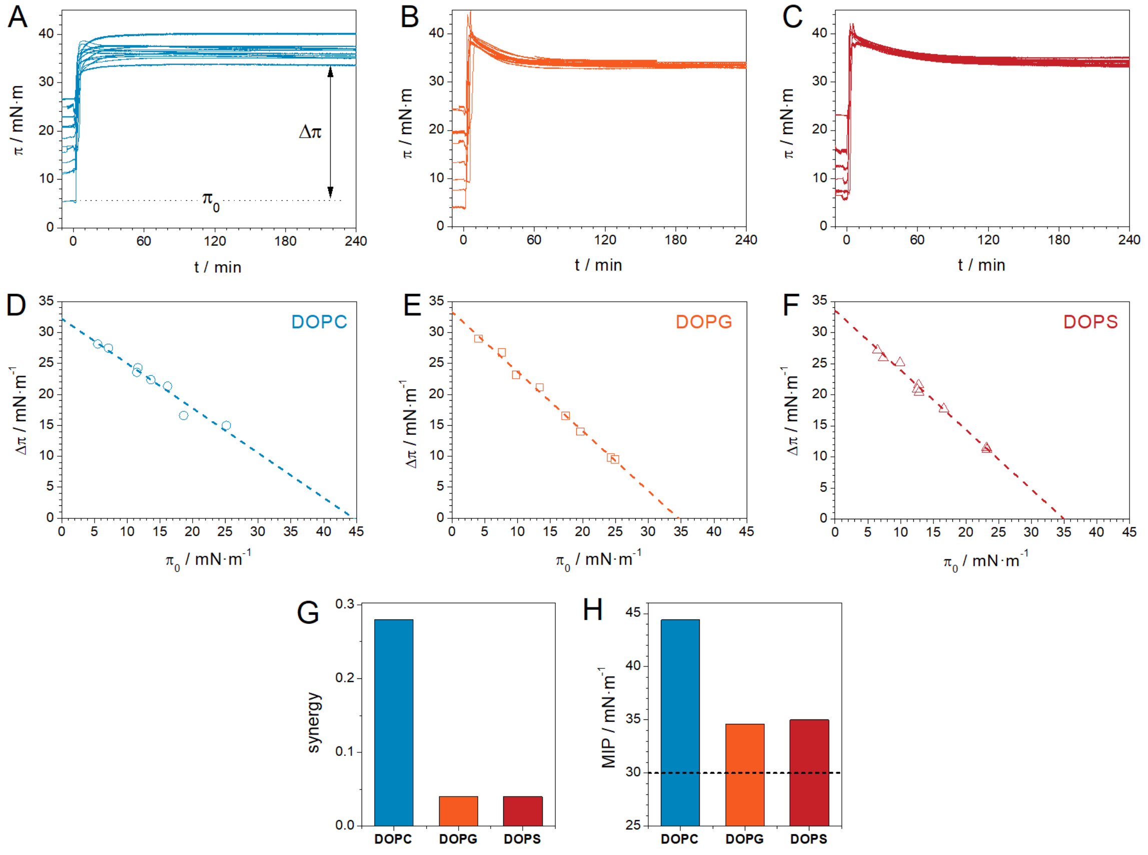

Figure S11. Adsorption of melittin to lipid monolayers at various initial surface pressures (π_0). The surface pressure (π) was measured over time after injection of Melittin into the subphase (PBS) at t=0. $\pi(t < 0)$ is the initial surface pressure (π_0) of the pure lipid monolayer. Changes in surface pressure ($\Delta\pi$) after equilibration of Melittin within the lipid monolayer evaluated and plotted as a function of π_0 (open symbols) together with a linear fit of the data (dotted lines). **(A)** Surface pressure upon Melittin adsorption to DOPC (blue). **(B)** Surface pressure upon Melittin adsorption to DOPG (orange). **(C)** Surface pressure upon Melittin adsorption to DOPS (red). **(D)** Changes in equilibrium surface pressure of DOPC (blue). **(E)** Changes in equilibrium surface pressure of DOPG (orange). **(F)** Changes in equilibrium surface pressure of DOPS (red). **(G)** The synergy parameter of the interaction of Melittin with the different lipids was determined from the slope of the linear fits (slope + 1) shown in panels D-E. The concentration of Melittin in the PBS subphase was 3.5 nM in all experiments. **(H)** The maximum insertion pressure (MIP) of Melittin in the different lipid monolayers was determined from the intersection of the linear fitting curves with the x-axis shown in panels D-E. The monolayer-bilayer equivalence pressure is indicated by a dotted line at π = 30 mN/m.

Supplementary Tables

Table S1. Composition, concentration, pore size of extrusion membranes, and collisional voltages applied of various liposome preparations.

lipid composition	size	final concentration	collisional voltage
DOPC	50 nm	0.5 mM	20 V – 200 V
DOPE	50 nm	0.5 mM	20 V – 200 V
DOPS	50 nm	0.5 mM	20 V – 200 V
DOPC/ DOPE/DOPS: cholesterol (5:2:2:1)	50 nm	0.5 mM	150 V
DOPC/DOPE/DOPS (5:2:2)	50 nm	0.5 mM	150 V
DOPC/ DOPE/DOPS/ cholesterol (5:2:2:1)	50 nm	5 mM	150 V
DOPC/ DOPE/DOPS/ cholesterol (5:2:2:1)	100 nm	0.5 mM	150 V
DOPC/ DOPE/DOPS/ cholesterol (5:2:2:1)	50 nm	0.5 mM	20 V – 200 V
**lipid composition proteoliposomes**	**size**	**final concentration**	**collisional voltage**
DOPG/DOPE (5:2)	100 nm	2.0 mM	50 V
DOPC/ DOPE/DOPS/ cholesterol (5:2:2:1)	100 nm	2.0 mM	50 V
DOPC/DOPE/PI(3)P (8:1:1)	100 nm	2.0 mM	50 V
DOPC/DOPE (8:2)	100 nm	2.0 mM	n/a

Table S2. Experimentally observed and theoretical mass-to-charge ratios (*m/z's)* of DOPC liposomes in native MS experiments (see Figure 2).

Composition	m/z experimental	m/z theoretical
$[DOPC_5]^{2+}$	1966.41	1964.49
$[DOPC_3]^{1+}$	2358.73	2357.79
$[DOPC_{10}]^{3+}$	2620.89	2619.65
$[DOPC_7]^{2+}$	2751.97	2750.58
$[DOPC_{11}]^{3+}$	2882.72	2881.52
$[DOPC_4]^{1+}$	3145.15	3143.38
$[DOPC_{13}]^{3+}$	3407.14	3405.24
$[DOPC_9]^{2+}$	3538.67	3536.18
$[DOPC_{14}]^{3+}$	3669.22	3667.11
$[DOPC_5]^{1+}$	3931.15	3928.97

Table S3. Experimentally observed and theoretical mass-to-charge ratios (*m/z's)* of DOPE liposomes in native MS experiments (see Figure S1).

Composition	m/z experimental	m/z theoretical
$[DOPE_3]^{1+}$	2231.82	2231.65
$[DOPE_4]^{1+}$	2975.34	2975.20
$[DOPE_9]^{2+}$	3346.99	3346.97
$[DOPE_5]^{1+}$	3718.34	3718.74
$[DOPE_{11}]^{2+}$	4091.47	4090.52
$[DOPE_6]^{1+}$	4462.95	4462.29

Table S4. Experimentally observed and theoretical mass-to-charge ratios (*m/z's)* of DOPS liposomes in native MS experiments (see Figure S1).

Composition	m/z experimental	m/z theoretical
$[DOPS_3]^{1+}$	2363.65	2363.84
$[DOPS_7]^{2+}$	2757.57	2757.64
$[DOPS_4]^{1+}$	3151.74	3151.44
$[DOPS_9]^{2+}$	3545.48	3545.25
$[DOPS_5]^{1+}$	3939.88	3939.05
$[DOPS_{16}]^{3+}$	4201.49	4201.59
$[DOPS_{11}]^{2+}$	4331.59	4332.86
$[DOPS_{17}]^{3+}$	4463.29	4464.13
$[DOPS_6]^{1+}$	4728.68	4726.66

Table S5. Experimentally observed and theoretical mass-to-charge ratios (m/z`s) of p40(phox) (P) with DOPC/DOPE/PI(3)P (8:1:1) liposomes in denaturing MS experiments (see Figure 4B).

Composition	m/z experimental	m/z theoretical
$[P]^{9+}$	1841.95	1834.06
$[DOPC_2 + DOPE_3]^{2+}$	1902.92	1901.92
$[DOPC_3 + DOPE_2]^{2+}$	1923.94	1922.94
$[DOPC_4 + DOPE]^{2+}$	1944.97	1943.97
$[DOPC_5]^{2+}$	1965.99	1964.99
$[P]^{8+}$	2071.95	2054.12
$[DOPC_3 + DOPE_3]^{2+}$	2296.22	2294.72
$[DOPC_4 + DOPE_2]^{2+}$	2316.75	2315.74
$[DOPC_5 + DOPE]^{2+}$	2337.77	2336.76
$[DOPC_6]^{2+}$	2358.60	2357.79

Table S6. Experimentally observed and theoretical mass-to-charge ratios (m/z`s) of p40(phox) (P) with DOPC/DOPE/PI(3)P (8:1:1) liposomes in native MS experiments (see Figure 4C).

Composition	m/z experimental	m/z theoretical
[DOPC$_5$]$^{2+}$	1967.15	1964.99
[P]$^{8+}$	2073.00	2054.12
[DOPC$_4$ + DOPE$_2$]$^{2+}$	2317.58	2315.74
[DOPC$_5$ + DOPE]$^{2+}$	2338.57	2336.76
[DOPC$_6$]$^{2+}$	2359.64	2357.79
[P]$^{7+}$	2368.58	2347.42

Table S7. Experimentally observed and theoretical mass-to-charge ratios (m/z`s) of Melittin (M) with DOPC/DOPE/DOPS/cholesterol liposomes in denaturing MS experiments (see Figure 5A).

Composition	m/z experimental	m/z theoretical
[M+DOPC+ lysoPE]$^{4+}$	1029.16	1029.10
[M+DOPC+ lysoPC]$^{4+}$	1039.68	1039.62
[lysoPC$_2$]$^{1+}$	1043.70	1043.70
[M+ lysoPC+lysoPE$_2$]$^{4+}$	1083.18	1082.87
[M+DOPC+ lysoPE$_2$]$^{4+}$	1093.84	1093.61
[M+ lysoPE]$^{3+}$	1109.69	1109.94
[M+ lysoPC]$^{3+}$	1123.71	1123.95
[M+ DOPE+lysoPC+lysoPE]$^{4+}$	1149.24	1148.93
[M+DOPC+ lysoPC+lysoPE]$^{4+}$	1159.75	1159.44
[M+DOPC]$^{3+}$	1212.12	1212.04
[DOPE+ lysoPE]$^{1+}$	1223.86	1223.86
[DOPE+lysoPC]$^{1+}$	1265.90	1265.90
[DOPC+ lysoDOPC]$^{1+}$	1307.95	1307.95

Table S8. Experimentally observed and theoretical mass-to-charge ratios (*m/z's)* of Melittin (M) with DOPC/DOPE/DOPS/cholesterol liposomes in native MS experiments (see Figure 5B).

Composition	m/z experimental	m/z theoretical
[M+ lysoDOPC]$^{3+}$	1123.57	1123.95
[M+ DOPE]$^{3+}$	1196.78	1198.02
[M+DOPC]$^{3+}$	1211.51	1212.03
[M$_3$]$^{7+}$	1222.84	1221.36
[M$_4$]$^{9+}$	1266.82	1266.55
[DOPC+ lysoDOPC]$^{1+}$	1307.58	1307.95

Supplementary References

1. Wang, L. & Tonggu, L. Membrane protein reconstitution for functional and structural studies. *Sci. China Life Sci.* **58**, 66–74 (2015).
2. Sobott, F., Hernández, H., McCammon, M. G., Tito, M. A. & Robinson, C. V. A tandem mass spectrometer for improved transmission and analysis of large macromolecular assemblies. *Anal. Chem.* **74**, 1402–7 (2002).
3. Hernández, H. & Robinson, C. V. Determining the stoichiometry and interactions of macromolecular assemblies from mass spectrometry. *Nat. Protoc.* **2**, 715 (2007).
4. Morgner, N. & Robinson, C. V. Massign: An Assignment Strategy for Maximizing Information from the Mass Spectra of Heterogeneous Protein Assemblies. *Anal. Chem.* **84**, 2939–2948 (2012).
5. Calvez, P., Bussières, S., Éric Demers & Salesse, C. Parameters modulating the maximum insertion pressure of proteins and peptides in lipid monolayers. *Biochimie* **91**, 718–733 (2009).
6. Calvez, P., Demers, E., Boisselier, E. & Salesse, C. Analysis of the contribution of saturated and polyunsaturated phospholipid monolayers to the binding of proteins. *Langmuir* **27**, 1373–1379 (2011).
